# Non-coding RNAs as regulators of chromosomal instability in breast cancer

**DOI:** 10.3389/fonc.2026.1894119

**Published:** 2026-08-19

**Authors:** Deisy Pinilla-Peña, Anlly Niño-Salgado, Milena Rondón-Lagos

**Affiliations:** School of Biological Sciences, Universidad Pedagógica y Tecnológica de Colombia, Tunja, Colombia

**Keywords:** breast cancer, chromosomal instability, DNA repair, genomic instability, non-coding RNAs, RNA dysregulation, tumor progression

## Abstract

Breast cancer is a highly heterogeneous disease characterized by extensive genomic and chromosomal instability (CIN), a hallmark that drives tumor evolution, intratumoral heterogeneity, therapeutic resistance, and poor clinical outcomes. Increasing evidence indicates that non-coding RNAs (ncRNAs) are important regulators of genome maintenance and chromosome stability. However, their specific contributions to CIN and the strength of the available evidence remain incompletely understood. This review examines the role of the major ncRNA classes, including circular RNAs, microRNAs, PIWI-interacting RNAs, small nucleolar RNAs, and long non-coding RNAs, in the regulation of CIN-related processes in breast cancer. We discuss the molecular mechanisms by which these ncRNAs regulate key pathways involved in CIN, while critically evaluating the strength of the experimental evidence supporting their functional roles. We also examine their associations with distinct breast cancer molecular subtypes and assess their potential as biomarkers and therapeutic targets, highlighting current limitations and knowledge gaps that hinder clinical translation. Collectively, the available evidence supports an emerging role for ncRNAs as regulators of CIN while underscoring the need for further mechanistic and subtype-specific studies to validate their clinical utility.

## 1 Introduction

Breast cancer (BC) is the most frequently diagnosed malignancy and the leading cause of cancer-related mortality among women worldwide, representing a heterogeneous group of tumors with distinct genetic, molecular, and clinical characteristics (1). BC development is influenced by a complex interplay of genetic, hormonal, and environmental factors, including age, family history, early menarche or menopause, obesity, alcohol consumption, and hormonal contraceptive use (2). According to the St. Gallen consensus, BC is classified into four major molecular subtypes—Luminal A, Luminal B, HER2-enriched, and triple-negative breast cancer (TNBC)—which exhibit distinct receptor profiles, biological behaviors, and therapeutic responses (3). However, in clinical practice, treatment decisions are primarily guided by immunohistochemistry-based surrogate subtypes defined by hormone receptor (HR) and HER2 status, with Ki-67 expression providing additional information for therapeutic stratification (4).

The intrinsic heterogeneity of BC and the limitations of current biomarkers have highlighted the need to identify additional molecular regulators involved in tumor evolution and genomic instability. In this context, RNA molecules have emerged as critical modulators of cancer biology and genome maintenance. Beyond their diagnostic and prognostic potential, RNAs influence CIN by controlling gene expression programs and pathways involved in DNA repair, cell-cycle progression, and chromosome segregation (5). Messenger RNAs (mRNAs), in addition to serving as templates for protein synthesis, can regulate gene expression through untranslated regions (UTRs) and other regulatory elements (6). Conversely, ncRNAs, including microRNAs (miRNAs) and long non-coding RNAs (lncRNAs), perform diverse regulatory functions that have expanded the traditional view of RNA biology beyond protein coding (7).

ncRNAs participate in essential processes related to genome maintenance, including DNA damage responses, chromatin organization, replication control, and mitotic fidelity. Their dysregulation can contribute to genomic instability (GI) and CIN by altering signaling pathways involved in proliferation, apoptosis, migration, metastasis, and therapeutic resistance (8). Depending on their molecular context and targets, ncRNAs may function as oncogenic or tumor-suppressive regulators, influencing tumor initiation and progression. Furthermore, advances in single-cell RNA sequencing have enabled the characterization of CIN-associated transcriptional states and intratumoral heterogeneity, providing new insights into tumor evolution and interactions with the tumor microenvironment (9).

This review examines the mechanisms through which ncRNAs influence GI and CIN in BC, highlighting their potential applications as diagnostic, prognostic, and therapeutic targets. As a narrative review, relevant literature was selected based on its contribution to understanding the molecular mechanisms of GI and CIN, the role of ncRNAs in genome maintenance and CIN, and their therapeutic implications in BC. Recent original research articles and comprehensive reviews were prioritized to provide an updated and integrative overview of GI mechanisms, CIN-associated processes, ncRNA biogenesis and functional diversity, including major ncRNA classes involved in BC biology, as well as emerging ncRNA-based therapeutic strategies and their combination with targeted and epigenetic therapies. Expanding our understanding of the complex interactions between ncRNAs, GI, and chromosomal evolution will be essential to uncover new molecular vulnerabilities and develop more precise strategies for BC diagnosis, prognosis, and treatment.

## 2 Types and mechanisms of GI

**Figure 1:**
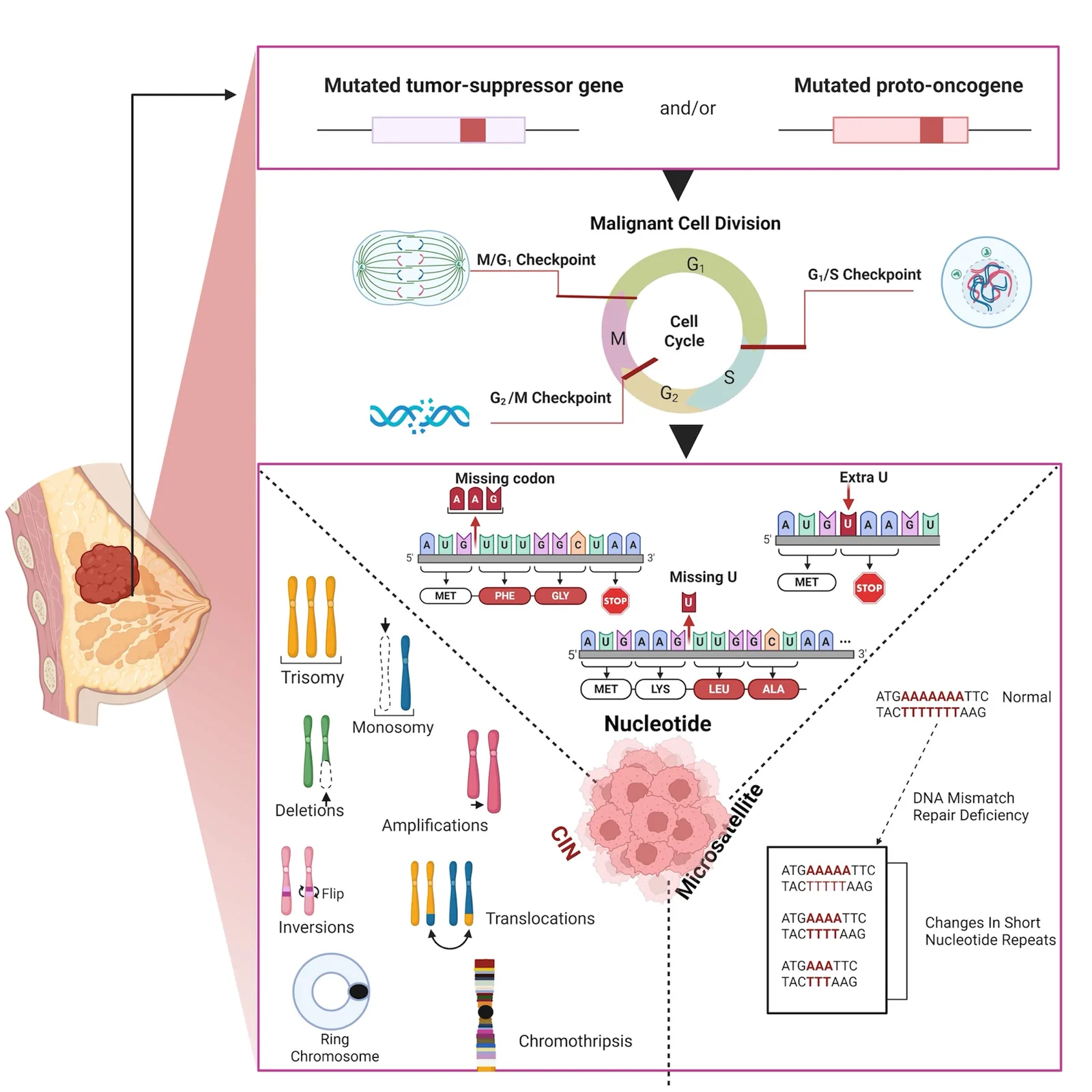
Genomic (GI) and chromosomal instability (CIN) in breast cancer (BC). Disruption of tumor suppressors and activation of proto-oncogenes impair cell-cycle control, driving abnormalities in proliferation, apoptosis, and survival. These changes include sequence-level alterations (deletions, insertions, substitutions) and chromosomal abnormalities (aneuploidy, translocations, amplifications, chromothripsis), along with microsatellite instability due to mismatch repair deficiency. Collectively, they promote malignant transformation and BC progression.

GI is a hallmark of cancer and a major driver of intra- and intertumoral genetic heterogeneity. It encompasses a broad spectrum of genetic alterations, ranging from single-nucleotide variants and small insertions or deletions to large-scale chromosomal rearrangements. The onset of GI is primarily driven by defects in genome maintenance mechanisms, including impaired DNA damage checkpoints, defective DNA repair pathways, replication stress, and compromised mitotic surveillance. Disruption of these processes impairs DNA replication fidelity, transcriptional regulation, and chromosome segregation, ultimately promoting catastrophic genomic events such as chromothripsis (10). Additional factors, including telomere attrition, centrosome amplification, persistent DNA damage, oncogenic mutations, and aneuploidy, further exacerbate GI, accelerating tumor evolution, intratumoral heterogeneity, and therapeutic resistance (11) (**Figure 1**).

GI is generally classified into three major forms: nucleotide instability, characterized by base substitutions, insertions, and deletions; microsatellite instability (MSI), resulting from alterations in short tandem repeats; and chromosomal instability (CIN), which comprises persistent numerical and structural chromosomal abnormalities (12).

## 3 CIN and its role in BC

CIN is characterized by the ongoing acquisition of numerical and structural chromosomal abnormalities resulting from defects in chromosome segregation and genome maintenance during cell division (13). CIN is broadly classified into whole-chromosome instability (W-CIN) and structural CIN. W-CIN involves the continuous gain or loss of whole chromosomes, leading to numerical abnormalities such as aneuploidy and contributing to both intra- and intertumoral heterogeneity (13). In contrast, structural CIN comprises chromosomal rearrangements, including deletions, insertions, inversions, translocations, and catastrophic events such as chromothripsis (13).

**Figure 2:**
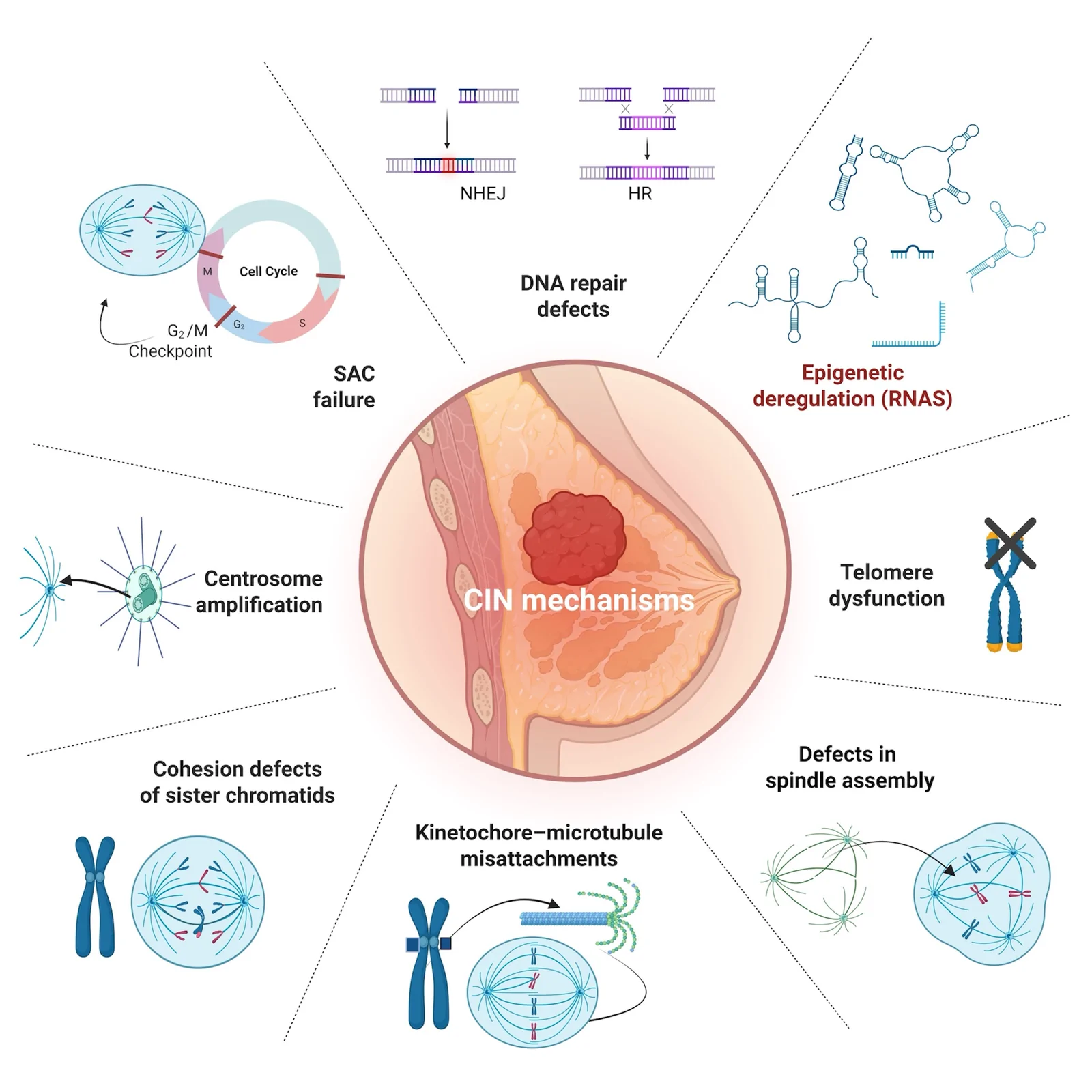
Mechanisms of chromosomal instability (CIN). CIN arises from defects in DNA repair, epigenetic regulation, telomere maintenance, and mitosis. Impaired HR and NHEJ allow persistent DNA double-strand breaks, while RNA-mediated dysregulation affects genes controlling the cell cycle and chromosome segregation. Telomere dysfunction triggers chromosome fusions and breakage–fusion–bridge cycles. Mitotic errors—including centrosome amplification, cohesion defects, and faulty kinetochore–microtubule attachments that evade spindle assembly checkpoint control—lead to chromosome missegregation. Together, these defects propagate structural and numerical chromosomal abnormalities across cell divisions. CIN, chromosomal instability; HR, homologous recombination; NHEJ, non-homologous end joining; ncRNAs, non-coding RNAs; SAC, spindle assembly checkpoint.

CIN results from defects in sister chromatid cohesion, kinetochore–microtubule attachment, spindle assembly checkpoint (SAC) signaling, DNA replication and repair, and telomere maintenance. These alterations promote chromosome missegregation and genome rearrangements, driving tumor evolution, therapeutic resistance, and poor clinical outcomes across multiple cancer types, including BC (13) (**Figure 2**).

In addition, aberrant expression or dysfunction of centromere and kinetochore proteins has emerged as a major contributor to CIN. These proteins, including CENP-A, HJURP, and components of the constitutive centromere-associated network (CCAN) and KMN complexes, are essential for spindle microtubule attachment, spindle assembly checkpoint (SAC) signaling, and faithful sister chromatid segregation. Their deregulation disrupts kinetochore integrity, leading to chromosome missegregation, aneuploidy, chromosome bridges, micronucleus formation, and genome rearrangements (14). Notably, centromere and kinetochore dysfunction also contribute to tumor progression, and tumors with extremely high CIN levels appear less tolerant to additional genotoxic stress, thereby influencing prognosis and responses to radiotherapy and chemotherapy (14).

Beyond protein-coding components, centromeric and pericentromeric regions are actively transcribed into diverse ncRNAs, including long non-coding RNAs and satellite repeat-derived transcripts, which are increasingly recognized as key regulators of chromosome biology rather than transcriptional by-products (15). These ncRNAs participate in centromeric chromatin organization, CENP-A deposition, kinetochore assembly, heterochromatin maintenance, and SAC regulation, thereby ensuring faithful chromosome segregation. Their dysregulation compromises these processes, promoting chromosome missegregation, aneuploidy, micronucleus formation, and CIN. Furthermore, aberrant accumulation of satellite-derived transcripts has been reported in multiple human cancers and is associated with genomic instability, tumor progression, and altered therapeutic responses, highlighting centromeric ncRNAs as emerging regulators of genome integrity and potential therapeutic targets (16–18).

Increasing evidence also indicates that ncRNAs, particularly lncRNAs, contribute to the regulation of CIN by modulating p53 signaling, centromere and kinetochore function, and mitotic checkpoint pathways, thereby linking epigenetic and transcriptional regulation to chromosome segregation, genome stability, and BC progression (15, 19).

## 4 Role of ribonucleic acids in the regulation of GI and CIN in BC

The maintenance of genomic integrity relies on a complex interplay between DNA- and RNA-mediated regulatory mechanisms. In BC, coding and ncRNAs have emerged as key modulators of GI and CIN, participating in the regulation of DNA damage responses, chromatin dynamics, and chromosome segregation. Understanding the biogenesis and functional diversity of ncRNAs is therefore critical for deciphering the molecular mechanisms linking GI and CIN to BC development and progression.

### 4.1 Biogenesis of ncRNAs

RNAs are key regulators of cellular physiology that extend far beyond their classical role as intermediaries between DNA and proteins. They are broadly classified into coding RNAs (mRNAs) and ncRNAs, the latter comprising structural RNAs, such as ribosomal RNAs (rRNAs) and transfer RNAs (tRNAs), as well as regulatory ncRNAs, including microRNAs (miRNAs), long non-coding RNAs (lncRNAs), circular RNAs (circRNAs), small non-coding RNAs (sncRNAs), PIWI-interacting RNAs (piRNAs), and small nucleolar RNAs (snoRNAs) (20, 21). These RNA classes are generated through distinct biogenesis pathways that determine their maturation, stability, subcellular localization, and biological functions.

Briefly, miRNAs are transcribed primarily by RNA polymerase II as primary transcripts (pri-miRNAs) and undergo sequential processing by the Drosha–DGCR8 complex in the nucleus and Dicer in the cytoplasm to generate mature miRNAs that associate with the RNA-induced silencing complex (RISC) (22, 23). Likewise, lncRNAs are mainly transcribed by RNA polymerase II and generally undergo capping, splicing, and polyadenylation, whereas circRNAs are generated through back-splicing events that produce highly stable covalently closed transcripts (24). In contrast, piRNAs originate from long single-stranded precursors through Dicer-independent pathways and interact with PIWI proteins to preserve genome integrity by repressing transposable elements (25). SnoRNAs are predominantly processed from introns of host genes and guide post-transcriptional modifications of ribosomal RNAs and other non-coding RNAs (26).

### 4.2 Types of ncRNAs and their relationship with GI and CIN in BC

**Figure 3:**
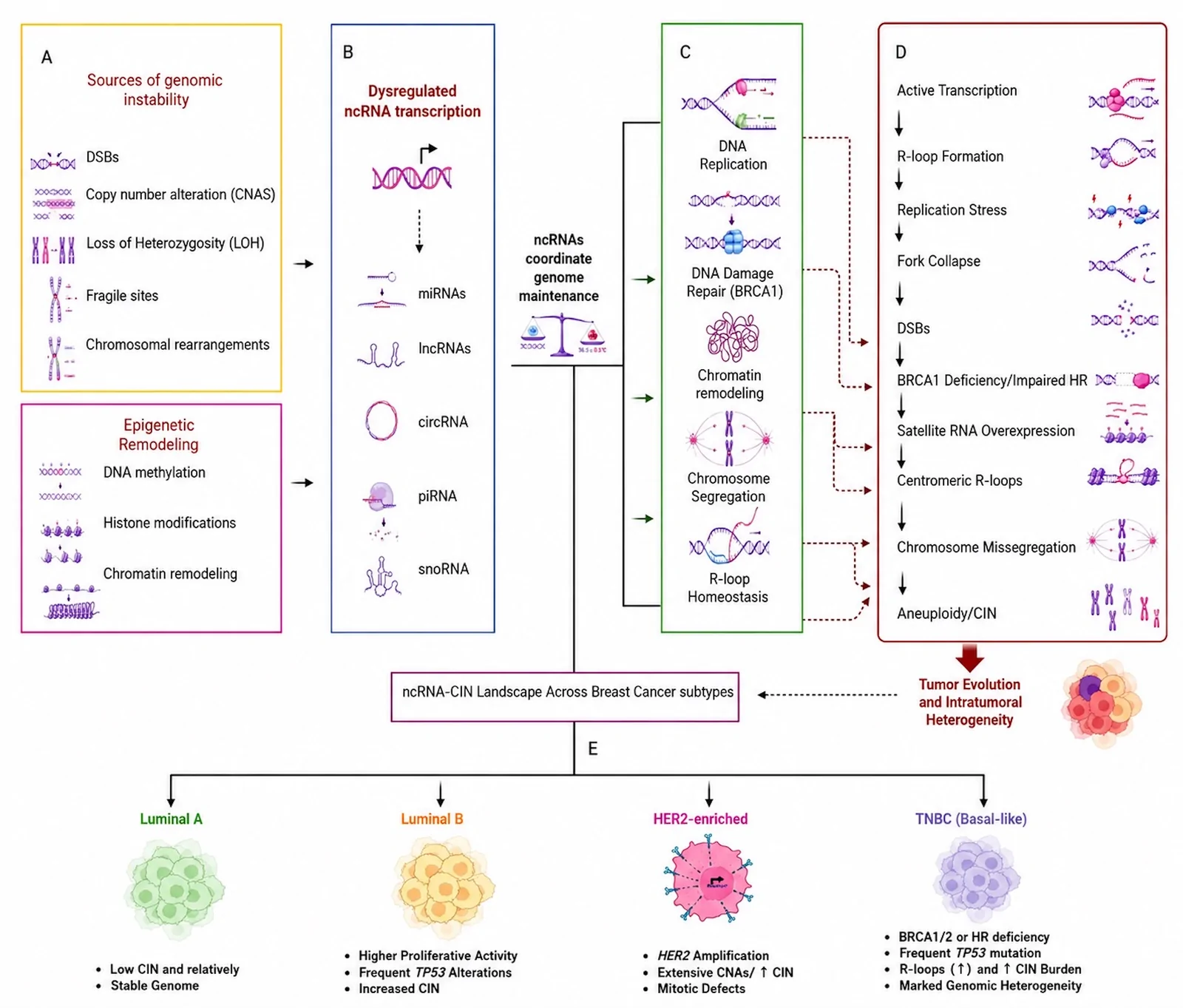
Integrated overview of the bidirectional relationship between genomic instability (GI), non-coding RNA dysregulation, and chromosomal instability (CIN) across breast cancer (BC) molecular subtypes. **(A)** GI, including DNA double-strand breaks (DSBs), copy number alterations (CNAs), loss of heterozygosity (LOH), fragile sites, and chromosomal rearrangements, together with epigenetic remodeling through aberrant DNA methylation, histone modifications, and chromatin remodeling, contributes to the dysregulation of non-coding RNA (ncRNA) expression in BC. **(B)** Altered expression of the major ncRNA classes, including microRNAs (miRNAs), long non-coding RNAs (lncRNAs), circular RNAs (circRNAs), PIWI-interacting RNAs (piRNAs), and small nucleolar RNAs (snoRNAs), influences key mechanisms involved in genome maintenance, including **(C)** DNA replication, DNA damage repair, chromatin remodeling, chromosome segregation, and R-loop homeostasis. **(D)** Conversely, defective genome maintenance and transcription-associated processes promote R-loop accumulation, replication stress, replication fork collapse, DNA double-strand breaks, homologous recombination (HR) deficiency, satellite RNA overexpression, centromeric R-loop formation, chromosome missegregation, aneuploidy, and ultimately CIN, thereby driving tumor evolution and intratumoral heterogeneity. **(E)** The biological consequences of ncRNA dysregulation occur within distinct molecular contexts across BC subtypes. Luminal A tumors generally exhibit low CIN and relatively stable genomes. Luminal B tumors display increased proliferative activity, frequent *TP53* alterations, and higher CIN. HER2-enriched tumors are characterized by *HER2* amplification, extensive copy number alterations, and mitotic defects. Whereas basal-like/triple-negative breast cancer (TNBC) is associated with BRCA1/2-related homologous recombination deficiency, frequent *TP53* mutations, increased R-loop accumulation, the highest CIN burden, and marked genomic heterogeneity.

Increasing evidence indicates that both, coding and ncRNAs, actively contribute to the maintenance of genomic integrity by regulating DNA replication, DNA damage repair, chromosome segregation, and chromatin organization. Among regulatory ncRNAs, circRNAs, miRNAs, piRNAs, snoRNAs and lncRNAs, have emerged as key modulators of these processes through distinct molecular mechanisms. In BC, the expression of these ncRNAs is profoundly influenced by GI and epigenetic remodeling. Numerous ncRNA genes are located within chromosomal regions frequently affected by copy number alterations, common fragile sites, or recurrent DNA double-strand breaks (DSBs), making them particularly susceptible to deregulation during tumor evolution (27, 28) (**Figure 3A**).

Specific genomic regions in BC are particularly susceptible to DNA damage, chromatin dysregulation, and epigenetic alterations, creating a permissive landscape for aberrant ncRNA expression. Early genomic studies demonstrated that numerous ncRNA loci are located within chromosomal regions frequently affected by loss of heterozygosity (LOH), copy number alterations, or recurrent chromosomal rearrangements in breast tumors (29, 30). These recurrent genomic alterations often target chromosomal regions harboring ncRNAs (**Figure 3B**), leading to changes in their expression through gene dosage imbalances, chromatin remodeling, or epigenetic deregulation. Collectively, these findings provide a mechanistic link between GI and ncRNA dysregulation, supporting the concept that structural and numerical chromosomal alterations can reshape the ncRNA landscape during BC initiation and progression (28).

Beyond structural genomic alterations, epigenetic dysregulation represents another major mechanism driving ncRNA deregulation in BC. Aberrant DNA methylation, altered histone modifications, chromatin remodeling, and changes in higher-order chromatin organization modify promoter accessibility, chromatin architecture, and transcriptional activity, thereby disrupting ncRNA expression independently of DNA sequence alterations (31) (**Figure 3A**). These epigenetic abnormalities not only alter the expression of established ncRNAs but may also promote the emergence of novel ncRNA transcripts and isoforms with context-dependent regulatory functions, reshaping the transcriptomic landscape and influencing DNA damage responses, intratumoral heterogeneity, and disease progression (32). Collectively, these findings highlight that both genomic and epigenetic alterations cooperate to remodel the ncRNA repertoire in BC, thereby contributing to GI, tumor evolution, and subtype-specific transcriptional programs (33).

Conversely, transcription-associated processes can themselves become an important source of GI and CIN. One of the principal mechanisms is the generation of DNA damage during active transcription. Indeed, transcription-associated DSBs represent a major source of GI (34), whereas the aberrant accumulation of R-loops—three-stranded nucleic acid structures composed of a DNA–RNA hybrid and a displaced single-stranded DNA—induces replication stress, replication fork collapse, and additional DSB formation, thereby promoting CIN (35) (**Figure 3C**). In response to DSBs, homologous recombination is activated, a repair pathway in which BRCA1 plays a central role in BC. Loss of BRCA1 function impairs homologous recombination, compromises genomic stability, and promotes the accumulation of oncogenic alterations. Beyond its canonical role in DNA repair, BRCA1 deficiency also induces satellite RNA overexpression, leading to replication fork destabilization, centromeric R-loop accumulation, chromosome missegregation, and aneuploidy, all of which contribute to the aggressive phenotype of many breast tumors (36) (**Figure 3D**).

Although the molecular mechanisms linking ncRNAs to GI described above are broadly relevant to BC, accumulating evidence indicates that ncRNA-mediated CIN is strongly influenced by molecular subtype (**Figure 3E**). The biological and clinical impact of ncRNA-mediated CIN appears to vary across BC subtypes. Luminal A tumors generally exhibit lower levels of CIN and relatively preserved genomic stability, whereas Luminal B tumors display increased proliferative activity, higher CIN, and a greater frequency of *TP53* alterations, which may contribute to subtype-specific ncRNA regulatory programs (37–40). HER2-enriched tumors are characterized by HER2 amplification, extensive copy-number alterations, and elevated CIN, providing a genomic context in which ncRNAs may modulate both, HER2 signaling and mitotic fidelity (40, 41). In contrast, basal-like/TNBC typically exhibit the highest CIN burden, frequent *TP53* mutations, homologous recombination deficiency, including BRCA1/2-associated defects, and marked genomic heterogeneity, all of which are closely linked to dysregulated ncRNA expression and function (41–43). These subtype-specific molecular landscapes suggest that ncRNA–CIN interactions are unlikely to be uniform across BC and underscore the importance of considering molecular subtype when investigating ncRNA-mediated mechanisms of GI and developing subtype-specific biomarkers and therapeutic strategies. However, direct comparative studies systematically evaluating ncRNA–CIN networks across all BC molecular subtypes remain limited. Consequently, current knowledge is uneven among subtypes, highlighting the need for comprehensive and standardized studies integrating cytogenomic, transcriptomic, and functional analyses to define subtype-specific ncRNA signatures and clarify their contribution to CIN.

Collectively, these findings highlight the bidirectional relationship between GI, epigenetic remodeling, and ncRNA dysregulation in BC. Genomic and epigenetic alterations reshape the ncRNA landscape, whereas dysregulated ncRNAs, in turn, regulate DNA replication, DNA repair, chromatin remodeling, chromosome segregation, and R-loop homeostasis, ultimately promoting chromosomal instability, tumor evolution, and subtype-specific disease progression, as summarized in **Figure 3**.

#### 4.2.1 Circular RNAs

**Figure 4:**
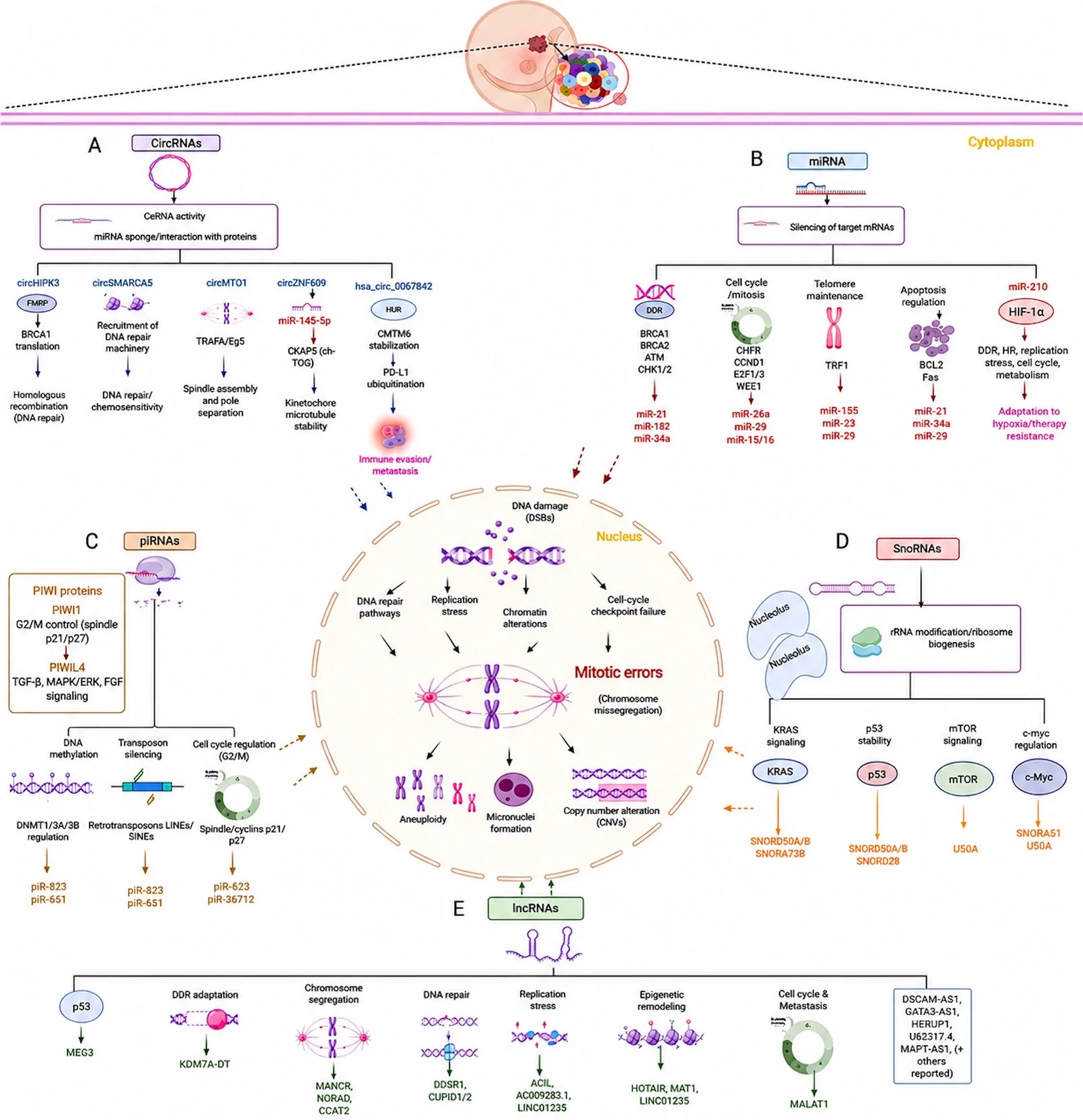
Non-coding RNA-mediated mechanisms contributing to chromosomal instability (CIN) in breast cancer (BC). **(A)** CircRNAs regulate genome maintenance through competing endogenous RNA (ceRNA) activity and protein interactions, influencing DNA repair, spindle assembly, chromosome segregation, microtubule–kinetochore stability, and immune evasion. **(B)** miRNAs modulate multiple pathways associated with CIN, including DNA damage response (DDR), cell-cycle progression, telomere maintenance, apoptosis, and adaptation to hypoxia, thereby influencing genome stability and tumor progression. **(C)** PIWI-interacting RNAs (piRNAs) and PIWI proteins contribute to genome integrity through DNA methylation, transposon silencing, and G2/M checkpoint control, while their dysregulation may promote genomic instability. **(D)** Small nucleolar RNAs (snoRNAs) regulate ribosome biogenesis, rRNA modification, nucleolar function, and signaling pathways involving KRAS, p53, mTOR and c-Myc, thereby modulating cellular processes associated with genome maintenance. **(E)** Long non-coding RNAs (lncRNAs) regulate DNA repair, replication stress, chromosome segregation, cell-cycle progression, and epigenetic remodeling through diverse molecular mechanisms that influence chromosomal stability and tumor evolution. Collectively, alterations in these ncRNA classes converge on pathways controlling DNA repair, replication stress, chromatin organization, mitotic fidelity, and cell-cycle checkpoints, ultimately promoting DNA double-strand breaks (DSBs), chromosome missegregation, micronuclei formation, copy number alterations (CNVs), aneuploidy, and chromosomal instability in BC.

Current evidence suggests that circRNAs may contribute to genomic stability and CIN in BC, through multiple regulatory mechanisms. CircRNAs function primarily as competitive endogenous RNAs (ceRNAs) that modulate gene expression by sequestering miRNAs and interacting with mRNAs. Beyond these functions, circRNAs regulate transcription, alternative splicing, and protein–RNA interactions, contributing to post-transcriptional homeostasis (44, 45). Through these mechanisms, circRNAs have emerged as important regulators of processes directly linked to CIN, including DNA repair, mitotic regulation, chromosome segregation, and tumor progression (**Figure 4A**).

Several circRNAs have been functionally associated with the maintenance of genomic stability in BC. For instance, circHIPK3 (11p13) promotes BRCA1 translation by competing with FMRP, thereby enhancing homologous recombination repair, preserving chromosomal stability, and increasing sensitivity to DNA-damaging agents (46). Similarly, circSMARCA5, derived from the *SMARCA5* gene located at chromosome 4q31.21, interacts with DNA exons and facilitates the recruitment of DNA repair machinery to damaged chromatin. This mechanism improves chemosensitivity, and its reduced circRNA-to-linear RNA ratio in patient blood samples suggests its potential utility as a liquid biopsy biomarker (45, 47). In addition, circMTO1 (6q13) contributes to accurate chromosome segregation by regulating spindle assembly and mitotic pole separation through the TRAF4/Eg5 pathway (48).

Likewise, circZNF609 (15q22.31) modulates CKAP5 (ch-TOG) expression through miR-145-5p sponging, promoting kinetochore–microtubule stability and reducing mitotic errors (49). More recently, hsa_circ_0067842, generated from exons 12–17 of the *SMC4* gene on chromosome 3, has been implicated in tumor adaptation by promoting metastasis and immune evasion through HuR cytoplasmic translocation, CMTM6 stabilization, and regulation of PD-L1 ubiquitination (50).

Collectively, these findings suggest that circRNAs can integrate miRNA/mRNA regulation, DNA repair pathways, mitotic organization, and immune-related mechanisms, highlighting their functional relevance in BC progression and their potential as diagnostic biomarkers and therapeutic targets.

However, the contribution of circRNAs to CIN appears to vary among BC molecular subtypes, although this concept remains incompletely understood and is supported mainly by correlative analyses, and indirect functional evidence rather than by systematic comparative studies. Luminal BCs (Luminal A and Luminal B) have been proposed to exhibit higher expression of tumor-suppressive circRNAs, including circASS1 (51), which may regulate cell-cycle progression and genome maintenance through miRNA sponge activity. Nevertheless, direct experimental evidence demonstrating that circASS1 preserves chromosomal stability or reduces CIN in these subtypes is currently lacking. Conversely, basal-like/TNBC, which generally display higher GI, have been associated with dysregulation of tumor-suppressive circRNAs and increased expression of oncogenic circRNAs, such as circGFRA1. This circRNA promotes BC progression by sequestering tumor-suppressive miRNAs and enhancing GFRA1-mediated activation of the PI3K/AKT signaling pathway (52). Although these effects may indirectly contribute to GI by stimulating uncontrolled proliferation and cell-cycle deregulation, a direct causal relationship between circGFRA1 and CIN has not yet been experimentally established.

Therefore, although specific circRNAs have demonstrated functional roles in pathways associated with genome maintenance, DNA damage response, and mitotic fidelity, subtype-specific circRNA–CIN relationships remain largely hypothetical. Current evidence supports the possibility that circRNAs contribute differently to genomic stability depending on the molecular context of BC. However, primary studies systematically evaluating the direct impact of individual circRNAs on CIN across BC subtypes are still required. Future comparative and functional investigations will be essential to determine whether circRNA alterations represent drivers, consequences, or modulators of CIN in distinct BC molecular backgrounds (24, 53, 54).

The circRNAs associated with CIN mechanisms across BC molecular subtypes are summarized in **Table 1**.

**Table 1:** ncRNAs associated with CIN mechanisms across BC molecular subtypes.

circRNA				
circRNA	Predominant BC subtypes	CIN-related mechanism	Functional relevance*	Evidence linking the circRNA to CIN**
circHIPK3	Breast cancer (subtype not fully defined); reported in BC models	Homologous recombination; DNA damage response	Indirect CIN-associated regulator	Mechanistic evidence
circSMARCA5	Breast cancer (subtype not fully defined)	DNA repair; chromatin regulation	Indirect CIN-associated regulator	Mechanistic evidence
circMTO1	Breast cancer (subtype not fully defined)	Spindle assembly; mitosis	Direct CIN regulator	Direct experimental evidence
circZNF609	Breast cancer (subtype not fully defined)	Kinetochore–microtubule attachment; chromosome segregation	Indirect CIN-associated regulator	Mechanistic evidence
hsa_circ_0067842	Breast cancer (subtype not fully defined)	Post-transcriptional regulation; immune signaling	Biomarker/Clinical association	Limited mechanistic evidence
circASS1	Luminal A and Luminal B breast cancers (proposed)	Cell-cycle regulation; proliferation control	Indirect CIN-associated regulator	Mechanistic evidence
circGFRA1	Basal-like/TNBC	Cell-cycle regulation; proliferation and tumor progression	Indirect CIN-associated regulator	Mechanistic evidence
General circRNA regulation	Subtype-dependent	RNA regulation; DNA repair; mitosis	Biomarker/Clinical association	No direct evidence linking to CIN
miRNA				
miRNA	Predominant BC subtypes	CIN-related mechanism	Functional relevance*	Evidence linking the miRNA to CIN**
miR-26a/26a-5p	TNBC	Mitotic defects, HRD, aneuploidy	Direct CIN regulator	Direct experimental evidence
miR-21	Luminal B, HER2+, TNBC	DDR/checkpoint impairment	Indirect CIN-associated regulator	Mechanistic evidence
miR-155	Luminal B, TNBC	Telomere dysfunction	Direct CIN regulator	Direct experimental evidence
miR-182	TNBC	BRCA1/2, ATM inhibition	Direct CIN regulator	Direct experimental evidence
miR-23 family	TNBC	Wnt/hypoxia pathways	Indirect CIN-associated regulator	Limited mechanistic evidence
miR-34a	Multiple	p53/BRCA axis, chromosome segregation	Direct CIN regulator	Direct experimental evidence
miR-15/16	Multiple	DDR, apoptosis	Direct CIN regulator	Direct experimental evidence
miR-29 family	Multiple	Cell-cycle and chromatin regulation	Indirect CIN-associated regulator	Mechanistic evidence
miR-99a	Luminal A	Cell-cycle regulation	Biomarker/Clinical association	No direct evidence linking to CIN
miR-30 family	Luminal A	Cell-cycle regulation	Biomarker/Clinical association	No direct evidence linking to CIN
miR-210	Basal-like/TNBC; hypoxic tumors	DNA damage response; homologous recombination; replication stress	Indirect CIN-associated regulator	Mechanistic evidence
miR-4728	HER2+	HER2 signaling	Biomarker/Clinical association	No direct evidence linking to CIN
piRNA/PIWI protein				
piRNA/PIWI protein	Predominant BC subtypes	CIN-related mechanism	Functional relevance*	Evidence linking the piRNA/PIWI protein to CIN**
PIWIL1	Multiple	Mitotic progression; spindle assembly	Direct CIN regulator	Direct experimental evidence
PIWIL4	Multiple	TGF-β/MAPK signaling	Indirect CIN-associated regulator	Mechanistic evidence
piR-36712	Luminal	DDR activation; epigenetic stability	Indirect CIN-associated regulator	Mechanistic evidence
piR-823	Basal-like/TNBC	DNMT activation; epigenetic remodeling	Indirect CIN-associated regulator	Mechanistic evidence
piR-651	Basal-like/TNBC	G2/M regulation	Indirect CIN-associated regulator	Mechanistic evidence
PIWI–piRNA pathway	Multiple	Transposon silencing; DNA methylation	Direct CIN regulator	Direct experimental evidence
snoRNA				
snoRNA	Predominant BC subtypes	CIN-related mechanism	Functional relevance*	Evidence linking the snoRNA to CIN**
SNORA73	Breast cancer (general)	Chromatin regulation; cancer stem cell differentiation	Indirect CIN-associated regulator	Mechanistic evidence
SNORA73B	Breast cancer (general)	Tumor progression; aggressive phenotype	Biomarker/Clinical association	Bioinformatic/Clinical association
U50A	Breast cancer (general)	Mitosis; mTOR/c-Myc signaling	Indirect CIN-associated regulator	Mechanistic evidence
SNORA51	Breast cancer (general)	Ribosome regulation; c-Myc signaling; proliferation	Indirect CIN-associated regulator	Mechanistic evidence
SNORD28	Breast cancer with p53 alterations	p53 pathway; DNA repair; cell proliferation	Indirect CIN-associated regulator	Mechanistic evidence
SNORD50A/B	HER2-enriched; basal-like/TNBC	KRAS signaling; p53 stability; cellular stress response	Indirect CIN-associated regulator	Mechanistic evidence
SNORD3A	Luminal A; Luminal B	rRNA maturation; ribosome assembly; translational fidelity	Biomarker/Clinical association	Associative evidence
SnoRNA loci alterations (general)	HER2-enriched; basal-like/TNBC	Ribosome biogenesis; genome maintenance; stress response	Biomarker/Clinical association	Associative evidence
lncRNA				
lncRNA	Predominant BC subtypes	CIN-related mechanism	Functional relevance*	Evidence linking the lncRNA to CIN**
MANCR	TNBC	Mitosis; cytokinesis	Direct CIN regulator	Direct experimental evidence
DDSR1	Multiple	Homologous recombination	Direct CIN regulator	Direct experimental evidence
ACIL	Multiple	ATR–Chk1; fork protection	Indirect CIN-associated regulator	Mechanistic evidence
NORAD	Multiple	Chromosome segregation	Direct CIN regulator	Direct experimental evidence
MEG3	Basal-like/TNBC	p53-mediated genome stability	Indirect CIN-associated regulator	Mechanistic evidence
CCAT2	Multiple	Chromosome missegregation	Direct CIN regulator	Direct experimental evidence
MAT1	Multiple	CNV-driven instability	Indirect CIN-associated regulator	Mechanistic evidence
DSCAM-AS1	Luminal A	ER-dependent transcription	Biomarker/Clinical association	Associative evidence
GATA3-AS1	Luminal B	Replication stress	Biomarker/Clinical association	Associative evidence
CUPID1/2	Luminal	HR/NHEJ regulation	Indirect CIN-associated regulator	Mechanistic evidence
HERUP1	HER2-enriched	Chromatin remodeling	Biomarker/Clinical association	Associative evidence
HOTAIR	HER2-enriched	Epigenetic remodeling	Indirect CIN-associated regulator	Mechanistic evidence
AC009283.1	HER2-enriched	Replication licensing	Indirect CIN-associated regulator	Mechanistic evidence
LINC01235	HER2-enriched	Replication stress	Indirect CIN-associated regulator	Mechanistic evidence
GAS5	Basal-like/TNBC	Genome maintenance	Biomarker/Clinical association	Associative evidence
LUCAT1	Basal-like/TNBC	DNA repair inhibition	Indirect CIN-associated regulator	Mechanistic evidence
MALAT1	Basal-like/TNBC	PI3K/AKT/Wnt activation	Indirect CIN-associated regulator	Mechanistic evidence
LINC00511	Basal-like/TNBC	Replication stress	Indirect CIN-associated regulator	Mechanistic evidence
U62317.4	High-CIN BC	p53/CDC20 signaling	Biomarker/Clinical association	Bioinformatic/Clinical association
MAPT-AS1	Multiple	Tumor progression	Biomarker/Clinical association	Bioinformatic/Clinical association
LINC00668	Multiple	Chemoresistance	Indirect CIN-associated regulator	Mechanistic evidence
AL138789.1	Multiple	Prognostic signature	Biomarker/Clinical association	Bioinformatic/Clinical
AC046168.1	Multiple	CIN-associated prognostic biomarker	Biomarker/Clinical association	Limited
KDM7A-DT	TP53-mutant/subtype-dependent BC	DNA damage response; genotoxic stress; copy number alterations	Indirect CIN-associated regulator	Limited mechanistic evidence

*Only ncRNAs for which published evidence suggests a potential relationship with CIN were included. The classification reflects the current level of experimental evidence. Many ncRNAs have been investigated primarily in relation to BC progression, prognosis, or therapeutic response, whereas direct functional evidence linking them to chromosome segregation defects, replication stress, DNA damage repair, or aneuploidy remains limited.

**Evidence classification: Direct experimental evidence refers to experimental studies demonstrating a direct role in CIN-related processes (e.g., chromosome segregation, mitosis, cytokinesis, or aneuploidy). Mechanistic evidence refers to experimental studies defining the molecular mechanism by which the ncRNA regulates genome stability pathways (e.g., DNA damage response [DDR], homologous recombination, replication stress, ATR–Chk1 signaling, or p53 pathways), although a direct effect on CIN has not always been demonstrated. Limited mechanistic evidence refers to experimental studies suggesting a potential role of the ncRNA in genome stability-related pathways; however, the underlying molecular mechanism is not fully characterized and direct effects on CIN have not been demonstrated. Associative evidence refers to studies showing associations with breast cancer molecular subtypes or CIN-related biological processes without direct mechanistic validation. Bioinformatic/Clinical association refers to evidence derived primarily from transcriptomic analyses, prognostic signatures, or clinical correlations.

The chromosomal loci and supporting references for each ncRNA are provided in the corresponding sections of the main text.

#### 4.2.2 MicroRNAs

MicroRNAs (miRNAs) are small non-coding RNAs that regulate gene expression at the post-transcriptional level and play fundamental roles in DNA damage response (DDR), cell cycle progression, apoptosis, chromatin remodeling, and genome maintenance. Their dysregulation is a hallmark of BC and contributes to GI and CIN through the coordinated regulation of these pathways (**Figure 4B**). Functionally, miRNAs involved in breast carcinogenesis can be broadly classified into oncogenic miRNAs (oncomiRs), which promote tumor initiation and progression by inhibiting tumor suppressor genes, and tumor-suppressive miRNAs, which preserve genomic integrity by regulating DNA repair pathways, mitotic fidelity, and apoptotic signaling.

The contribution of miRNAs to CIN also appears to differ across BC molecular subtypes, reflecting their distinct genomic landscapes and biological characteristics. Luminal A tumors, which generally exhibit lower levels of CIN, are enriched in tumor-suppressive miRNAs such as miR-99a and members of the miR-30 family. Although these miRNAs regulate cell proliferation and cell-cycle progression, their direct role in preserving chromosomal stability has not yet been demonstrated (30, 55). Conversely, Luminal B, HER2-enriched, and basal-like/TNBC display progressively higher levels of GI and harbor distinct miRNA expression profiles that may differentially influence CIN-related pathways. For example, amplification of the *HER2* locus in HER2-enriched tumors results in the co-expression of miR-4728, which modulates HER2 signaling and resistance to anti-HER2 therapies, although its direct contribution to CIN remains unclear (56). Overall, current evidence supports a subtype-dependent contribution of miRNAs to GI. However, systematic comparative studies evaluating miRNA–CIN networks across all BC molecular subtypes remain scarce.

Hypoxia-responsive miRNAs further reinforce the subtype-specific contribution of miRNAs to GI related pathways in BC. Among them, miR-210 is one of the best-characterized hypoxamiRs and is consistently induced by HIF-1α under hypoxic conditions, particularly in aggressive BC subtypes. Experimental evidence indicates that miR-210 regulates DDR, homologous recombination, replication stress, cell-cycle progression, and mitochondrial metabolism, thereby promoting adaptation to hypoxic stress, therapeutic resistance, and tumor progression. Although direct evidence demonstrating that miR-210 induces chromosome segregation defects or CIN is still lacking, its central role in genome maintenance pathways suggests that it may contribute indirectly to GI in hypoxic breast tumors (57, 58).

Among the best-characterized oncomiRs involved in CIN in BC, are miR-26a/miR-26a-5p, miR-21, miR-155, miR-182, and the miR-23 family, all of which promote GI through distinct mechanisms affecting DNA repair, mitotic fidelity, telomere maintenance, and oncogenic signaling pathways (8). Collectively, these oncomiRs disrupt multiple mechanisms required for the preservation of genome integrity, thereby facilitating chromosomal alterations, tumor evolution, and disease progression.

Of these oncomiRs, miR-26a promotes CIN by disrupting mitosis and cytokinesis through the inhibition of CHFR, LARP1, and YWHAE, leading to replication stress, centrosome abnormalities, multipolar spindle formation, chromosome mis-segregation, and aneuploidy (59–61). Restoration of CHFR, and to a lesser extent YWHAE, rescues these mitotic defects, supporting their role as critical mediators of miR-26a-induced CIN (60). Its mature form, miR-26a-5p, further promotes homologous recombination deficiency (HRD) by negatively regulating BARD1 and NABP1, sensitizing BC cells to DNA-damaging agents while enhancing cisplatin-induced apoptosis through Fas receptor- and mitochondrial-mediated pathways (59, 60). Consistent with these functions, miR-26a-5p has been associated with a BRCAness phenotype in TNBC, highlighting its subtype-specific relevance in homologous recombination-deficient tumors (59). Additionally, although miR-26a-1 is located within the frequently deleted 3p21.1 region in epithelial malignancies, whether loss of this locus directly contributes to CIN remains unknown (62).

Similarly, miR-21 (17q23.1/17q23.2) impairs DNA repair and cell-cycle checkpoints, promoting G2/M arrest and increasing the accumulation of genomic alterations, whereas its inhibition enhances apoptosis following radiotherapy (63). Although miR-21 is frequently overexpressed in aggressive BC subtypes, including Luminal B, HER2-positive, and basal-like/TNBC tumors, current evidence supports its role in tumor progression and therapeutic resistance more strongly than a subtype-specific effect on CIN.

Likewise, miR-155 (21q21.3) contributes to CIN by destabilizing telomeres through the regulation of TRF1 (64). Its overexpression in Luminal B and particularly in basal-like/TNBC tumors further suggests that telomere dysfunction may contribute to the elevated CIN characteristic of these subtypes (64). miR-182 (7q32.2) promotes GI by suppressing key DDR genes, including *BRCA1, BRCA2*, and *ATM*, thereby impairing homologous recombination repair and facilitating the accumulation of chromosomal alterations (65). Elevated miR-182 expression has been reported predominantly in TNBC and BRCA1-deficient tumors, supporting its potential contribution to subtype-specific DNA repair defects and CIN.

Finally, the miR-23 family, comprising the miR-23a–27a–24–2 cluster on chromosome 19 and the miR-23b–27b–24–1 cluster on chromosome 9 (C9orf3), regulates hypoxia and Wnt signaling through VHL and APC. Loss of miR-23b/27b promotes uncontrolled proliferation and contributes to CIN (66). Although aberrant expression of this family has been associated with aggressive BC phenotypes, including TNBC, its direct contribution to subtype-specific CIN remains to be established.

Conversely, several tumor-suppressive miRNAs play essential roles in preserving genome integrity in BC by coordinating DDR, cell-cycle progression, apoptosis, chromosomal segregation, and epigenetic homeostasis. Loss or functional impairment of these miRNAs disrupts multiple mechanisms required for accurate genome maintenance, thereby facilitating CIN, tumor progression, and therapeutic resistance. Among the best-characterized tumor-suppressive miRNAs are the miR-34a, miR-15/16, and miR-29 families (8, 67).

The miR-34a family (1p36.22) maintains genomic stability through activation of the p53 pathway and regulation of multiple downstream targets involved in cell-cycle progression, apoptosis, epithelial-to-mesenchymal transition (EMT), and DNA repair, including BCL2, SIRT1, EZH2, Twist1, CHK1, WEE1, and BMI1. In particular, miR-34a acts through the p53/BRCA1/BRCA2 axis to coordinate DNA repair and mitotic progression. Consequently, loss of miR-34a promotes chromosome segregation defects, deregulation of E2F1/E2F3, increased CIN, and reduced chemosensitivity. Similarly, the miR-15/16 family (13q14.3) enhances DDR efficiency and therapeutic response by regulating genes involved in cell-cycle control, apoptosis, and genome maintenance (68). Although reduced expression of miR-34a and miR-15/16 has been reported in aggressive BC subtypes, including TNBC, direct evidence linking their subtype-specific dysregulation to differences in CIN remains limited.

Another important tumor-suppressive group is the miR-29 family (miR-29a, miR-29b, and miR-29c), which regulates chromatin organization, epigenetic remodeling, cellular metabolism, and metastatic dissemination, thereby contributing to multiple processes involved in BC progression (69). Although oncogenic functions have been described in specific cancer contexts, the majority of evidence supports a tumor-suppressive role for the miR-29 family in BC, reflecting its context-dependent regulation of multiple molecular targets and signaling pathways (69). Among its members, miR-29a restrains cell proliferation by inducing G0/G1 cell-cycle arrest. In the context of CIN, this tumor-suppressive miRNA contributes to the maintenance of cellular homeostasis by regulating key cell-cycle pathways involved in genome integrity. Accordingly, downregulation of miR-29a increases the expression of proliferative regulators, including B-Myb, Cyclin D1, and Cyclin A2, thereby promoting uncontrolled cell growth, facilitating cell-cycle deregulation, and increasing the likelihood of accumulating genomic alterations that drive tumor evolution and cancer progression (70, 71).

In addition, members of the miR-29 family regulate signaling axes such as GATA3–miR-29b, EZH2–miR-29b/miR-30d–LOXL4, and TIMP3/STAT1/FOXO1, further supporting their role in controlling proliferation, invasion, and metastasis (69) With respect to genomic stability, current evidence suggests that miR-29a may contribute indirectly to genome maintenance by regulating cell-cycle pathways involved in cellular homeostasis. Accordingly, reduced miR-29a expression may facilitate the accumulation of genomic alterations associated with tumor progression (71). However, unlike miR-26a, miR-155, or miR-182, direct evidence demonstrating a causal role of the miR-29 family in CIN remains limited. Therefore, further functional studies are needed to clarify its contribution to CIN, BC evolution, and its potential utility as a diagnostic, prognostic, and therapeutic biomarker.

The miRNAs associated with CIN mechanisms across BC molecular subtypes are summarized in **Table 1**.

#### 4.2.3 PIWI-interacting RNAs

piRNAs constitute a highly conserved class of small non-coding RNAs that, together with PIWI proteins, preserve genome stability by silencing transposable elements and regulating DNA methylation through epigenetic mechanisms. Disruption of the PIWI–piRNA pathway compromises these functions, leading to the accumulation of DSBs, chromosomal rearrangements, aneuploidy, and ultimately GI and CIN (**Figure 4C**) (72). Beyond safeguarding genome integrity, this pathway also regulates somatic gene expression through interactions with Polycomb complexes and other epigenetic regulatory networks (73).

In BC, PIWI proteins further contribute to these processes in a subtype-dependent manner. PIWIL4 (11q21) promotes proliferation, migration, and tumor progression by activating the TGF-β, MAPK/ERK, and FGF signaling pathways (74) whereas PIWIL1 (12q24.33) regulates the G2/M transition through modulation of cyclins and the CDK inhibitors p21 and p27. Experimental inhibition of PIWIL1 disrupts mitotic spindle assembly, induces metaphase abnormalities, and promotes chromosome missegregation and CIN, although its centrosomal functions, described in colorectal cancer, remain to be elucidated in BC (75).

At the piRNA level, emerging evidence suggests subtype-specific functions. In luminal tumors, the tumor-suppressive piR-36712 contributes to genome maintenance by enhancing DNA damage responses and preserving epigenetic repression of transposable elements, while also repressing SEPW1 through competitive interactions with miR-7 and miR-324, thereby activating the p53/p21 axis and suppressing malignant phenotypes (76). Conversely, more aggressive subtypes, particularly basal-like and TNBC, are characterized by increased expression of oncogenic piRNAs such as piR-823 and piR-651. piR-823 promotes DNMT1, DNMT3A, and DNMT3B expression, resulting in aberrant DNA methylation, hypermethylation of the APC promoter, activation of Wnt/β-catenin signaling, and enhanced stemness and tumorigenicity (77). Likewise, dysregulation of piR-651 affects G2/M progression, and its inhibition induces cell-cycle arrest, highlighting the importance of piRNA-mediated regulation for faithful mitotic progression networks (73).

Although direct evidence linking these oncogenic piRNAs to CIN in BC remains limited, their ability to remodel the epigenetic landscape, alter DNA damage responses, and disrupt cell-cycle control strongly suggests that they indirectly contribute to GI and chromosome missegregation, particularly in biologically aggressive BC subtypes.

The piRNA/PIWI proteins associated with CIN mechanisms across BC molecular subtypes are summarized in **Table 1**.

#### 4.2.4 Small nucleolar RNAs

snoRNAs have emerged as important regulators of ribosome biogenesis, rRNA modification, and cellular stress responses, processes that may influence genome stability and CIN through the regulation of translational fidelity, replication stress, and tumor suppressor pathways (**Figure 4D**) (26, 78).

In BC, alterations in snoRNA expression and genomic integrity of snoRNA loci, may contribute to tumor evolution by affecting ribosomal function, cellular differentiation, proliferation, and mechanisms involved in genome maintenance (79). Several snoRNAs have been functionally implicated in CIN-related processes. For instance, SNORA73, which associates with chromatin, contributes to CIN by sustaining differentiation blocks in cancer stem cells, whereas its variant SNORA73B (1p35), specifically detected in BC, correlates with aggressive tumor characteristics and has been proposed as a potential early diagnostic biomarker (80, 81). Conversely, the tumor-suppressive snoRNA U50A (6q15) prolongs mitosis, reduces colony formation, and inhibits the mTOR pathway through c-Myc suppression, thereby increasing therapeutic sensitivity (82). In contrast, SNORA51 (chromosome 20) promotes stem cell-like properties by regulating the RPL3/NPM1/c-Myc axis, altering the nucleolar and nucleoplasmic distribution of RPL3 and NPM1 to enhance proliferation and survival (83). The interaction between snoRNAs and the p53 pathway further highlights their relevance in genome maintenance. SNORD28 (chromosome 14), which is repressed by p53, generates miRNA-like molecules that regulate TAF9B, influencing DNA repair and cell proliferation, while SNORD50A/B (6q14.3) regulate p53 stability through GMPS and TRIM21, contributing to preservation of genomic integrity. Notably, deletion of SNORD50A/B in p53-mutant contexts produces opposite effects, emphasizing the context-dependent role of these snoRNAs in tumorigenesis (78, 84).

In Luminal A and Luminal B BCs, specific members of the SNORD family, such as SNORD3A, have been associated with regulation of rRNA maturation and ribosome assembly, processes that may cooperate with ER-dependent transcriptional programs to maintain translational fidelity and limit metabolic or replication-associated stress (26, 85). Conversely, HER2-enriched and basal-like/TNBC, which frequently exhibit higher levels of GI, are associated with recurrent genomic alterations affecting snoRNA loci, including loss of the tumor-suppressive snoRNAs SNORD50A and SNORD50B. Deletion of SNORD50A/B disrupts their interaction with KRAS and contributes to enhanced oncogenic signaling and reduced p53 stabilization, promoting cellular proliferation and stress responses that may facilitate genome instability (86).

Collectively, these findings suggest that snoRNAs integrate ribonucleoprotein biogenesis, mTOR, c-Myc, KRAS, and p53 signaling networks, thereby regulating processes associated with CIN, differentiation, and tumor progression. However, although several snoRNAs have demonstrated functional effects on pathways linked to genome maintenance, direct experimental evidence showing that specific snoRNA alterations drive CIN, such as increased aneuploidy or chromosome segregation defects, remains limited. Therefore, current evidence supports a role for snoRNAs as modulators of CIN-associated mechanisms in BC, with potential subtype-specific effects, but further functional and comparative studies are required to establish their direct contribution to chromosomal instability and their applicability as biomarkers or therapeutic targets (26, 79).

The snoRNAs associated with CIN mechanisms across BC molecular subtypes are summarized in **Table 1**.

#### 4.2.5 Long non-coding RNAs

lncRNAs are key regulators of genome stability, modulating chromatin organization, epigenetic homeostasis, DDR, DNA repair, replication stress, chromosome segregation, and mitotic progression through epigenetic, transcriptional, and post-transcriptional mechanisms (**Figure 4E**). Increasing evidence supports a bidirectional interplay between lncRNAs and CIN in BC, whereby lncRNAs both, regulate and are dysregulated by GI, contributing to tumor progression, therapeutic resistance, and poor prognosis (87, 88). Consequently, their dysregulation compromises nuclear architecture and chromosomal organization, promoting DSBs, chromosome missegregation, aneuploidy, intratumoral heterogeneity, and CIN (89–91). These findings have identified several CIN-associated lncRNAs as promising biomarkers and therapeutic targets in BC (92, 93).

Mechanistically, lncRNAs regulate CIN through DNA repair, replication stress, mitotic checkpoint control, chromosome segregation, and epigenetic remodeling. Genome-protective lncRNAs include MANCR (10p15.1), which is required for faithful mitosis and cytokinesis, as its depletion induces chromosomal fragmentation and apoptosis (94); DDSR1 (12q23.3), which recruits BRCA1 and hnRNPUL1 to DSBs, facilitating homologous recombination repair (95); ACIL (chromosome 17), which stabilizes replication forks through ATR–Chk1 signaling under genotoxic stress (96); NORAD (20q11.23), which preserves chromosome segregation fidelity by sequestering Pumilio proteins, although its persistent overexpression may also enhance tumor aggressiveness through the TGF-β/RUNX2 pathway (91, 97, 98); and the tumor suppressor MEG3 (14q), which promotes genomic stability by enhancing p53 activity while inhibiting MDM2 and NF-κB signaling (19).

Conversely, oncogenic lncRNAs such as CCAT2 promote chromosome segregation errors and CIN (87), whereas MAT1 and LINC01235 contribute to GI through copy number variation (CNV)-driven oncogenic activity and epigenetic remodeling, respectively (88, 99). Similarly, MALAT1 (11q13) promotes cell-cycle progression and metastasis by activating the PI3K/AKT/mTOR and Wnt/β-catenin pathways and functioning as a competing endogenous RNA for miR-561-3p to upregulate TOP2A (100, 101). In addition, the estrogen-responsive lncRNAs CUPID1/2 (11q13) regulate the balance between homologous recombination (HR) and non-homologous end joining (NHEJ) by modulating enhancer-promoter looping at the CCND1 locus (102).

Emerging evidence further indicates that lncRNA expression is subtype-specific in BC. Luminal tumors preferentially express DSCAM-AS1, which contributes to luminal differentiation and ER-positive tumor programs (103), whereas GATA3-AS1 is enriched in luminal tumors and promotes proliferation through regulation of GATA3-associated transcriptional networks (104). In HER2-enriched BC, lncRNAs such as HOTAIR, HERUP1, AC009283.1, and LINC01235 have been implicated in chromatin regulation, transcriptional reprogramming, and tumor progression (105). Although these mechanisms may influence genome stability-associated processes, direct evidence linking these lncRNAs to replication stress, DNA damage accumulation, or CIN remains limited.

Consistently, AC009283.1 and LINC01235 further amplify CIN by upregulating replication licensing factors (MCM, CDC6, and CDT1), leading to DNA breaks, micronuclei formation, and clonal evolution in HER2-positive tumors (99, 106).

In contrast, basal-like and TNBC subtypes exhibit altered expression of several lncRNAs, including reduced levels of tumor-suppressive lncRNAs such as GAS5 and MEG3, and increased expression of oncogenic lncRNAs such as LUCAT1, MALAT1, and LINC00511. These alterations have been associated with regulation of cell proliferation, stemness, PI3K/AKT/mTOR and Wnt/β-catenin signaling pathways, and tumor progression, although their direct contribution to CIN and genome instability remains incompletely defined (107–109).

Additional CIN-associated lncRNAs, including U62317.4, MAPT-AS1, LINC00668, AL138789.1, AC002456.1, and AC046168.1, have been associated with high-CIN tumors, tumor progression, chemoresistance, or poor prognosis (110–115).

Recent evidence has further identified KDM7A-DT as a subtype-associated lncRNA linking genotoxic stress to GI-related pathways in BC (116). KDM7A-DT expression is regulated in a *TP53* mutation-dependent manner and is associated with copy number gain, aneuploidy, homologous recombination defects, and activation of DDR pathways across distinct BC subtypes. Mechanistically, KDM7A-DT inhibits TP53BP1-mediated DNA damage repair, promotes G2/M checkpoint arrest, and reduces apoptosis, thereby favoring the persistence of DNA damage and adaptation to genotoxic stress. Clinically, elevated KDM7A-DT expression correlates with aggressive tumor features, genomic alterations, and poor outcomes in basal-like/TNBC and luminal A tumors, supporting its role as a subtype-dependent regulator of genome stability-associated pathways, although direct evidence linking KDM7A-DT to chromosome segregation defects or CIN remains to be established.

Collectively, these findings indicate that lncRNAs act as integrative hubs linking the p53, ATR–Chk1, TGF-β, PI3K/AKT/mTOR, and Wnt/β-catenin pathways with DNA repair, replication stress, chromatin remodeling, and mitotic control, thereby sustaining CIN and tumor adaptability while highlighting their potential as biomarkers and therapeutic targets in BC, although the evidence remains uneven across individual lncRNAs and molecular subtypes (90, 91).

The lncRNAs associated with CIN mechanisms across BC molecular subtypes are summarized in **Table 1**.

## 5 Therapeutic strategies targeting ncRNAs associated with CIN in BC

Therapeutic strategies targeting ncRNAs have emerged as promising approaches to counteract CIN and tumor progression in BC. Because ncRNAs regulate key CIN-associated processes, including DDR, replication stress, mitotic checkpoint control, chromatin remodeling, and chromosome segregation, they represent attractive molecular targets for precision oncology (117, 118). Several ncRNA-directed interventions have been developed to restore genome stability, inhibit oncogenic signaling, and enhance tumor sensitivity to conventional therapies.

### 5.1 ncRNA-based therapeutic approaches in CIN-Driven BC

miRNA-based therapeutic strategies, including miRNA mimics and antagomiRs, have emerged as promising approaches to restore aberrant miRNA expression patterns associated with cancer progression and CIN (119, 120). These strategies aim either to replenish tumor suppressor miRNAs frequently downregulated during breast tumorigenesis or to inhibit oncogenic miRNAs that promote genomic instability, uncontrolled proliferation, and therapy resistance (121). Through modulation of downstream targets, miRNA-based interventions can restore pathways involved in DNA repair, apoptosis, cell-cycle regulation, and maintenance of genomic integrity.

**Figure 5:**
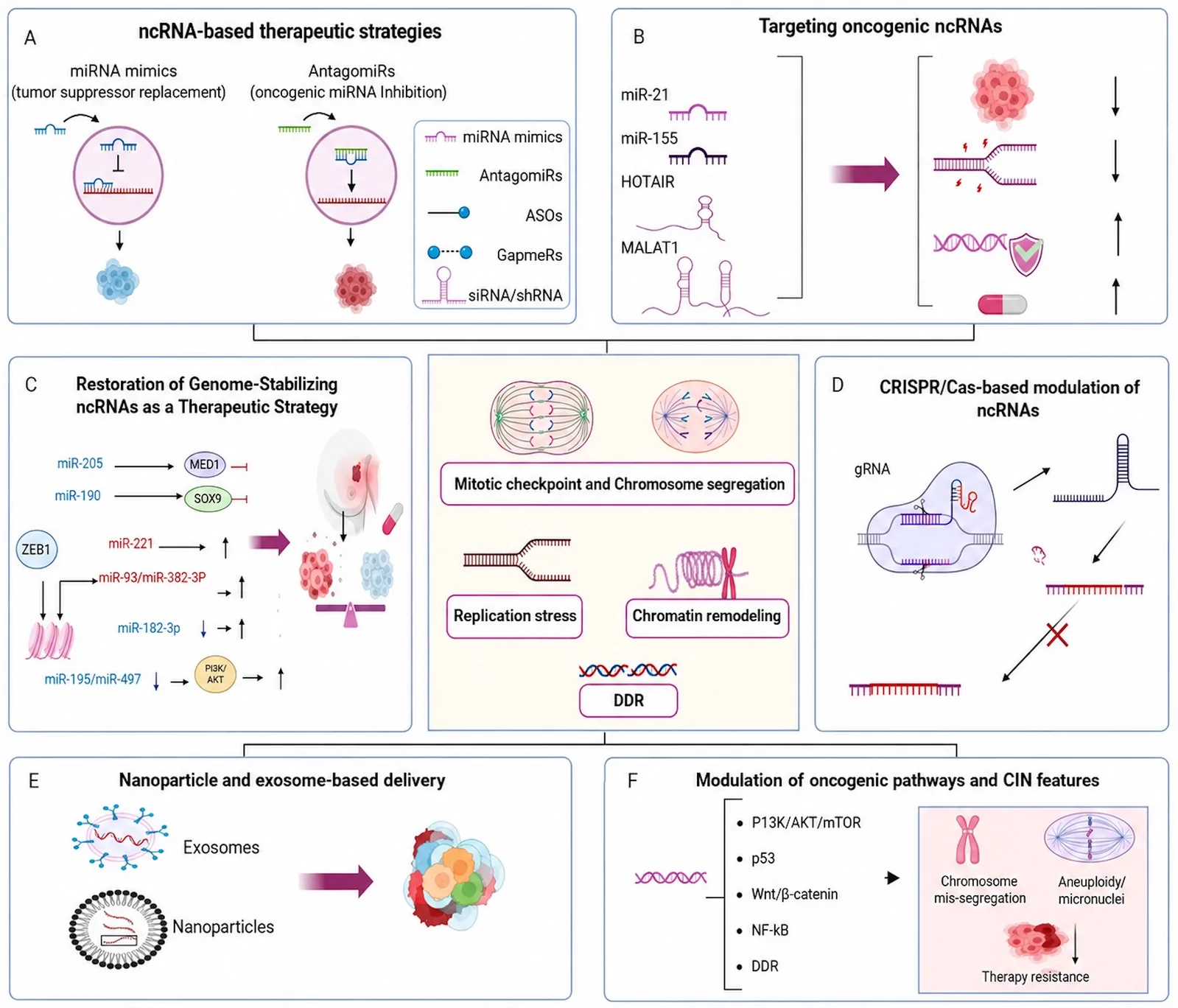
Therapeutic strategies targeting ncRNAs associated with chromosomal instability (CIN) in breast cancer (BC). **(A)** Overview of ncRNA-based therapeutic strategies used to target CIN in BC, including miRNA mimics, antagomiRs, antisense oligonucleotides (ASOs), GapmeRs, and siRNA/shRNA-based approaches. **(B)** Targeting oncogenic ncRNAs, such as miR-21, miR-155, HOTAIR, and MALAT1, to reduce tumor proliferation, replication stress, and genomic instability while improving DNA repair and therapeutic responsiveness. **(C)** Restoration of Genome-Stabilizing ncRNAs as a Therapeutic Strategy. Representative examples include miR-205-mediated suppression of MED1 and miR-190 targeting SOX9 to enhance tamoxifen sensitivity, as well as dysregulation of miR-221, miR-93, miR-382-3p, miR-182-3p, and ZEB1-mediated silencing of miR-195/497 contributing to endocrine resistance through activation of PI3K/AKT signaling. **(D)** CRISPR/Cas-based approaches for direct modulation, silencing, or knockout of ncRNAs implicated in CIN and tumor progression. **(E)** Nanoparticle- and exosome-mediated delivery systems designed to enhance intracellular stability, tumor specificity, and therapeutic efficacy of ncRNA-targeted agents. **(F)** Modulation of major CIN-associated signaling pathways, including PI3K/AKT/mTOR, p53, Wnt/β-catenin, NF-κB, and DNA damage response (DDR) pathways, resulting in reduced chromosome mis-segregation, aneuploidy, micronuclei formation, and therapy resistance.

Synthetic miRNA mimics have been developed to restore the activity of tumor suppressor miRNAs lost during tumor progression (122). Conversely, antagomiRs, antisense oligonucleotides (ASOs), Gapmer antisense oligonucleotides (GapmeRs; specialized ASOs designed to induce RNase H-mediated degradation of target RNAs, particularly lncRNAs), and siRNA/shRNA-based approaches have been primarily used to silence oncogenic ncRNAs, including dysregulated miRNAs, lncRNAs, and circRNAs involved in CIN-associated pathways (123) (**Figure 5A**). The therapeutic relevance of these strategies has been demonstrated in different BC subtypes, including HER2-positive BC and TNBC, where targeting specific miRNAs has resulted in reduced tumor growth and increased sensitivity to chemotherapy and targeted therapies (124–126).

In particular, inhibition of oncogenic ncRNAs such as miR-21, miR-155, HOTAIR (chromosome 12), and MALAT1 has been associated with reduced replication stress, improved DNA repair fidelity, suppression of tumor proliferation, and enhanced response to PARP inhibitors and chemotherapy, highlighting their potential as therapeutic targets in CIN-driven BC (72, 127) (**Figure 5B**).

Beyond oncogenic ncRNAs, restoration of genome-stabilizing ncRNAs may represent an alternative therapeutic strategy. For example, re-expression of NORAD, a lncRNA involved in maintaining chromosomal stability, may contribute to limiting tumor evolution in highly unstable breast tumors (128). Conversely, dysregulation of miR-221 (Xp11.3), miR-93 (7q22.1), and miR-382-3p (14q32), together with downregulation of miR-182-3p (7q32.2), has been associated with tamoxifen resistance and disease recurrence (129, 130). Moreover, ZEB1, a major regulator of epithelial–mesenchymal transition (EMT) in BC, promotes endocrine resistance through epigenetic silencing of miR-195 and miR-497 at 17p13.1, resulting in activation of the PI3K/AKT pathway (131) (**Figure 5C**).

### 5.2 Emerging technologies for ncRNA modulation and delivery

Recent advances in CRISPR/Cas technologies have further expanded the therapeutic landscape by enabling direct modulation, knockout, or reprogramming of ncRNAs implicated in CIN and tumor evolution (125) (**Figure 5D**). In parallel, nanoparticle- and exosome-based delivery systems have improved the intracellular stability, tumor specificity, and therapeutic efficiency of ncRNA-targeted agents in BC models (125, 126, 132) (**Figure 5E**). These platforms may overcome major limitations associated with RNA-based therapies, including poor stability, rapid degradation, and off-target effects.

Therapeutic targeting of ncRNAs may also indirectly modulate major oncogenic pathways associated with CIN, including PI3K/AKT/mTOR, p53, Wnt/β-catenin, NF-κB, and DDR signaling pathways, thereby reducing chromosome missegregation, aneuploidy, micronuclei formation, and therapy resistance (8) (**Figure 5F**).

### 5.3 Combination strategies involving ncRNAs, targeted therapies, and epigenetic drugs

Combination therapies involving miRNAs and targeted therapeutic agents have been extensively explored as strategies to enhance treatment response and overcome drug resistance in BC. In ER-positive BC, several miRNAs regulate tamoxifen responsiveness through modulation of key oncogenic pathways. For example, restoration of miR-205 expression reduces tamoxifen resistance by suppressing Mediator Subunit 1 (MED1), a transcriptional coactivator amplified or overexpressed in approximately 40–60% of BC cases (133). Similarly, miR-190 enhances tamoxifen sensitivity by targeting SOX9, a transcription factor with oncogenic functions in BC (134).

Increasing evidence also suggests that combining epigenetic therapeutics with ncRNA-based strategies may represent a promising approach for BC treatment. Epigenetic drugs, particularly DNA methyltransferase inhibitors (DNMTis) and histone deacetylase inhibitors (HDACis), can restore the expression of epigenetically silenced tumor suppressor genes, enhance sensitivity to chemotherapy, endocrine therapy, targeted therapies, and immunotherapy, and overcome therapeutic resistance in BC (135–142).

Conversely, ncRNA-based therapies, including miRNA mimics, antisense oligonucleotides, siRNAs, and lncRNA-targeting approaches, provide complementary strategies to restore tumor suppressor networks or inhibit oncogenic transcripts, although their clinical translation remains limited by challenges related to delivery, stability, and target specificity (123, 143, 144).

Given the extensive crosstalk between epigenetic regulation and ncRNA-mediated gene expression, combining these approaches may simultaneously reverse aberrant epigenetic states and reprogram dysregulated transcriptional networks, thereby enhancing therapeutic efficacy, reducing tumor heterogeneity, and overcoming treatment resistance (145). Although this combinatorial strategy remains largely at the preclinical stage, it represents a promising avenue for the development of more effective and personalized treatments for BC.

Collectively, ncRNA-based therapeutic strategies represent an emerging approach for targeting CIN-driven BC progression and improving patient outcomes. Considering that ncRNA expression is frequently regulated by epigenetic mechanisms, integrated therapeutic strategies combining epigenetic modulation and ncRNA targeting could provide novel opportunities to restore deregulated gene regulatory networks, overcome therapeutic resistance, and improve BC treatment. However, despite their considerable therapeutic promise, most of these approaches remain at the preclinical or early translational stage. Therefore, further mechanistic and translational studies are required to elucidate the molecular mechanisms underlying these interactions, validate their safety and efficacy, and determine their feasibility for effective clinical implementation in BC.

## 6 Conclusions

ncRNAs have emerged as key regulators of genome stability-associated processes in BC, expanding our understanding of the molecular mechanisms underlying CIN and tumor evolution. Once considered transcriptional by-products, they are now recognized as critical modulators of chromatin organization, DNA damage response and repair, replication stress, cell-cycle progression, chromosome segregation, and epigenetic regulation. Through these interconnected pathways, ncRNAs can influence the development of GI, aneuploidy, structural chromosomal alterations, and tumor adaptation. Nevertheless, the evidence supporting these functions remains heterogeneous, ranging from direct functional validation to mechanistic, associative, or bioinformatic/clinical evidence depending on the specific ncRNA.

Integrating the chromosomal localization of ncRNAs with genomic and cytogenetic data may provide valuable insights into how ncRNA dysregulation contributes to fragile genomic regions, copy-number alterations, structural variants, and CIN during BC progression. Future studies should also incorporate subtype-specific analyses together with single-cell, spatial, and multi-omics approaches to better define the context-dependent roles of ncRNAs in Luminal A, Luminal B, HER2-enriched, and basal-like/TNBC tumors, as well as their contribution to intratumoral heterogeneity and treatment response.

Although ncRNAs represent promising biomarkers and therapeutic targets, important challenges remain in establishing their causal role in CIN and validating their clinical utility. Addressing these gaps will require rigorous functional studies, standardized experimental methodologies, larger and well-characterized clinical cohorts, and integrated cytogenomic and transcriptomic analyses. Advancing our understanding of ncRNA-mediated regulation of GI and CIN will facilitate the development of clinically relevant biomarkers and more precise therapeutic strategies for patients with BC.

## References

[1] Nolan E Lindeman GJ Visvader JE . Deciphering breast cancer: from biology to the clinic. Cell. (2023) 186:1708–28. doi: 10.1016/j.cell.2023.01.040 36931265

[2] Sun YS Zhao Z Yang ZN Xu F Lu HJ Zhu ZY . Risk factors and preventions of breast cancer. Int J Biol Sci. (2017) 13:1387–97. doi: 10.7150/ijbs.21635 29209143 PMC5715522

[3] Goldhirsch A Winer EP Coates AS Gelber RD Piccart-Gebhart M Thürlimann B . Personalizing the treatment of women with early breast cancer: highlights of the st gallen international expert consensus on the primary therapy of early breast cancer 2013. Ann Oncol. (2013) 24:2206–23. doi: 10.1093/annonc/mdt303 23917950 PMC3755334

[4] Castellarnau-Visús M Soveral I Regueiro Espín P Pineros Manzano J Del Río Holgado M . Prognostic factors in luminal B-like HER2-negative breast cancer tumors. Surg Oncol. (2023) 49:101968. doi: 10.1016/J.SURONC.2023.101968 37364335

[5] Shaikh M Doshi G . Unraveling non-coding RNAs in breast cancer: mechanistic insights and therapeutic potential. Med Oncol. (2025) 42:37. doi: 10.1007/s12032-024-02589-x 39730979

[6] Li J Liu C . Coding or noncoding, the converging concepts of RNAs. Front Genet. (2019) 10:496. doi: 10.3389/fgene.2019.00496 31178900 PMC6538810

[7] Lozano-Villada S Puthanveettil SV . Noncoding RNAs orchestrating the central dogma. J Biol Chem. (2026) 302:110933. doi: 10.1016/j.jbc.2025.110933 41242393 PMC12753233

[8] Mohapatra S Winkle M Ton AN Nguyen D Calin GA . The role of non-coding RNAs in chromosomal instability in cancer. J Pharmacol Exp Ther. (2023) 384:10–9. doi: 10.1124/jpet.122.001357 36167417 PMC9827503

[9] Meléndez-Flórez MP Ortega-Recalde O Rangel N Rondón-Lagos M . Chromosomal instability and clonal heterogeneity in breast cancer: from mechanisms to clinical applications. Cancers (Basel). (2025) 17:122. doi: 10.3390/cancers17071222 40227811 PMC11988187

[10] Duijf PHG Nanayakkara D Nones K Srihari S Kalimutho M Khanna KK . Mechanisms of genomic instability in breast cancer. Trends Mol Med. (2019) 25:595–611. doi: 10.1016/j.molmed.2019.04.004 31078431

[11] Ferguson LR Chen H Collins AR Connell M Damia G Dasgupta S . Genomic instability in human cancer: molecular insights and opportunities for therapeutic attack and prevention through diet and nutrition. Semin Cancer Biol. (2015) 35:S5–S24. doi: 10.1016/J.SEMCANCER.2015.03.005 25869442 PMC4600419

[12] Yun D Yang Z . Identification of a four-lncRNA prognostic signature for colon cancer based on genome instability. J Oncol. (2021) 2021:7408893. doi: 10.1155/2021/7408893 34594379 PMC8478558

[13] Castellanos G Valbuena DS Pérez E Villegas VE Rondón-Lagos M . Chromosomal instability as enabling feature and central hallmark of breast cancer. Breast Cancer: Targets Ther. (2023) 15:189–211. doi: 10.2147/BCTT.S383759 36923397 PMC10010144

[14] Weiguo Z Jian-Hua M Wei Z Anshu K Ke L James B . Centromere and kinetochore gene misexpression predicts cancer patient survival and response to radiotherapy and chemotherapy. Nature. (2016) 7:1–15. doi: 10.1038/ncomms12619 27577169 PMC5013662

[15] Ideue T Tani T . Centromeric non-coding RNAs: conservation and diversity in function. Non Coding RNA. (2020) 6:4. doi: 10.3390/ncrna6010004 31963472 PMC7151564

[16] McNulty SM Sullivan L Sullivan BA . Human centromeres produce chromosome-specific and array-specific alpha satellite transcripts that are complexed with CENP-A and CENP-C. Dev Cell. (2017) 42:226–240.e6. doi: 10.1016/j.devcel.2017.07.001 28787590 PMC5568664

[17] Rošić S Köhler F Erhardt S . Repetitive centromeric satellite RNA is essential for kinetochore formation and cell division. J Cell Biol. (2014) 207:335–49. doi: 10.1083/jcb.201404097 25365994 PMC4226727

[18] Hall LE Mitchell SE O’Neill RJ . Pericentric and centromeric transcription: a perfect balance required. Chromosome Res. (2012) 20:535–46. doi: 10.1007/s10577-012-9297-9 22760449

[19] Song J Cui Q Gao J . Roles of lncRNAs related to the p53 network in breast cancer progression. Front Oncol. (2024) 14:1453807. doi: 10.3389/fonc.2024.1453807 39479021 PMC11521785

[20] Mattick JS Makunin IV . Non-coding RNA. Hum Mol Genet. (2006) 15:R17–29. doi: 10.1093/hmg/ddl046 16651366

[21] Goodall GJ Wickramasinghe VO . RNA in cancer. Nat Rev Cancer. (2021) 21:22–36. doi: 10.1038/s41568-020-00306-0 33082563

[22] Ha M Kim V . Regulation of microRNA biogenesis. Nat Rev Mol Cell Biol. (20145) 15:509–24. doi: 10.1038/nrm3838 25027649

[23] Treiber T Treiber N Meister G . Regulation of microRNA biogenesis and function. Thromb Haemost. (2012) 107:605–10. doi: 10.1160/TH11-12-0836 22318703

[24] Kristensen LS Andersen MS Stagsted LVW Ebbesen KK Hansen TB Kjems J . The biogenesis, biology and characterization of circular RNAs. Nat Rev Genet. (2019) 20:675–91. doi: 10.1038/s41576-019-0158-7 31395983

[25] Ozata DM Gainetdinov I Zoch A O'Carroll D Zamore PD . PIWI-interacting RNAs: small RNAs with big functions. Nat Rev Genet. (2019) 20:89–108. doi: 10.1038/s41576-018-0073-3 30446728

[26] Bratkovič T Božič J Rogelj B . Functional diversity of small nucleolar RNAs. Nucleic Acids Res. (2020) 48:1627–51. doi: 10.1093/nar/gkz1140 31828325 PMC7038934

[27] Calin GA Sevignani C Dumitru CD Hyslop T Noch E Yendamuri S . Human microRNA genes are frequently located at fragile sites and genomic regions involved in cancers. Proc Natl Acad Sci USA. (2004) 101:2999–3004. doi: 10.1073/pnas.0307323101 14973191 PMC365734

[28] Croce CM . Causes and consequences of microRNA dysregulation in cancer. Nat Rev Genet. (2009) 10:704–14. doi: 10.1038/nrg2634 19763153 PMC3467096

[29] Volinia S Calin GA Liu CG Ambs S Cimmino A Petrocca F . A microRNA expression signature of human solid tumors defines cancer gene targets. Proc Natl Acad Sci USA. (2006) 103:2257–61. doi: 10.1073/pnas.0510565103 16461460 PMC1413718

[30] Iorio MV Ferracin M Liu CG Veronese A Spizzo R Sabbioni S . MicroRNA gene expression deregulation in human breast cancer. Cancer Res. (2005) 65:7065–70. doi: 10.1158/0008-5472.CAN-05-1783 16103053

[31] Esteller M . Non-coding RNAs in human disease. Nat Rev Genet. (2011) 12:861–74. doi: 10.1038/nrg3074 22094949

[32] Frankel LB Christoffersen NR Jacobsen A Lindow M Krogh A Lund AH . Programmed cell death 4 (PDCD4) is an important functional target of the microRNA miR-21 in breast cancer cells. J Biol Chem. (2008) 283:1026–33. doi: 10.1074/jbc.M707224200 17991735

[33] Di Leva G Gasparini P Piovan C Ngankeu A Garofalo M Taccioli C . MicroRNA cluster 221-222 and estrogen receptor α interactions in breast cancer. J Natl Cancer Inst. (2010) 102:706–21. doi: 10.1093/jnci/djq102 20388878 PMC2873185

[34] Gómez-González B Dutta A Feng W . Editorial: The role of RNA in genome stability: to wreck or repair? Front Mol Biosci. (2022) 9:848217. doi: 10.3389/fmolb.2022.848217 35224000 PMC8865954

[35] Castillo-Guzman D Chédin F . Defining R-loop classes and their contributions to genome instability. DNA Repair (Amst). (2021) 106:103182. doi: 10.1016/J.DNAREP.2021.103182 34303066 PMC8691176

[36] Leclerc S Kitagawa K . The role of human centromeric RNA in chromosome stability. Front Mol Biosci. (2021) 8:642732. doi: 10.3389/fmolb.2021.642732 33869284 PMC8044762

[37] Perou CM Sørlie T Eisen MB van de Rijn M Jeffrey SS Rees CA . Molecular portraits of human breast tumours. Nature. (2000) 406:747–52. doi: 10.1038/35021093 10963602

[38] Naidoo K Pinder SE . Immunohistochemistry for triple-negative breast cancer. Methods Mol Biol. (2016) 1406:29–44. doi: 10.1007/978-1-4939-3444-7_3 26820943

[39] Curtis C Shah SP Chin SF Turashvili G Rueda OM Dunning MJ . The genomic and transcriptomic architecture of 2,000 breast tumours reveals novel subgroups. Nature. (2012) 486:346–52. doi: 10.1038/nature10983 22522925 PMC3440846

[40] The Cancer Genome Atlas Network . Comprehensive molecular portraits of human breast tumours. Nature. (2012) 490:61–70. doi: 10.1038/nature11412 23000897 PMC3465532

[41] Bakhoum SF Cantley LC . The multifaceted role of chromosomal instability in cancer and its microenvironment. Cell. (2018) 174:1347–60. doi: 10.1016/j.cell.2018.08.027 30193109 PMC6136429

[42] Turner N Reis-Filho JS . Basal-like breast cancer and the BRCA1 phenotype. Oncogene. (2006) 25:5846–53. doi: 10.1038/sj.onc.1209876 16998499

[43] Denkert C Liedtke C Tutt A von Minckwitz G . Molecular alterations in triple-negative breast cancer—the road to new treatment strategies. Lancet. (2017) 389:2430–42. doi: 10.1016/S0140-6736(16)32454-0 27939063

[44] Chen H Xie G Luo Q Yang Y Hu S . Regulatory miRNAs, circRNAs and lncRNAs in cell cycle progression of breast cancer. Funct Integr Genomics. (2023) 23:233. doi: 10.1007/s10142-023-01130-z 37432486

[45] Misir S Yaman SO Petrović N Sumer C Hepokur C Aliyazicioglu Y . circRNAs in drug resistance of breast cancer. Oncol Res. (2022) 30:157–72. doi: 10.32604/or.2022.027547 37304411 PMC10208077

[46] Grelloni C Garraffo R Setti A Rossi F Peruzzi G Cinquanta M . BRCA1 levels and DNA-damage response are controlled by the competitive binding of circHIPK3 or FMRP to the BRCA1 mRNA. Mol Cell. (2024) 84:4079–4094.e10. doi: 10.1016/j.molcel.2024.09.016 39389065

[47] Xu X Zhang J Tian Y Gao Y Dong X Chen W . CircRNA inhibits DNA damage repair by interacting with host gene. Mol Cancer. (2020) 19:128. doi: 10.1186/s12943-020-01246-x 32838810 PMC7446195

[48] Liu Y Dong Y Zhao L Su L Luo J . Circular RNA-MTO1 suppresses breast cancer cell viability and reverses monastrol resistance through regulating the TRAF4/Eg5 axis. Int J Oncol. (2018) 53:1752–62. doi: 10.3892/ijo.2018.4485 30015883

[49] Rossi F Beltran M Damizia M Grelloni C Colantoni A Setti A . Circular RNA ZNF609/CKAP5 mRNA interaction regulates microtubule dynamics and tumorigenicity. Mol Cell. (2022) 82:75–89.e9. doi: 10.1016/J.MOLCEL.2021.11.032 34942120 PMC8751636

[50] Li J Dong X Kong X Wang Y Li Y Tong Y . Circular RNA hsa_circ_0067842 facilitates tumor metastasis and immune escape in breast cancer through HuR/CMTM6/PD-L1 axis. Biol Direct. (2023) 18:48. doi: 10.1186/s13062-023-00397-3 37592296 PMC10436663

[51] Hou JC Xu Z Zhong SL Zhang HD Jiang LH Chen X . Circular RNA circASS1 is downregulated in breast cancer cells MDA-MB-231 and suppresses invasion and migration. Epigenomics. (2019) 11:199–213. doi: 10.2217/epi-2017-0167 30657346

[52] He R Liu P Xie X Zhou Y Liao Q Xiong W . circGFRA1 and GFRA1 act as ceRNAs in triple-negative breast cancer by regulating miR-34a. J Exp Clin Cancer Res. (2017) 36:145. doi: 10.1186/s13046-017-0614-1 29037220 PMC5644184

[53] Ghafouri-Fard S Hussen BM Taheri M Ayatollahi SA . Emerging role of circular RNAs in breast cancer. Pathol Res Pract. (2021) 227:153496. doi: 10.1016/j.prp.2021.153496 34052769

[54] Verduci L Strano S Yarden Y Blandino G . The circRNA–microRNA code: emerging implications for cancer diagnosis and treatment. Mol Oncol. (2019) 13:669–80. doi: 10.1002/1878-0261.12468 30719845 PMC6441890

[55] Lowery AJ Miller N Devaney A McNeill RE Davoren PA Lemetre C . MicroRNA signatures predict oestrogen receptor, progesterone receptor and HER2/neu receptor status in breast cancer. Breast Cancer Res. (2009) 11:R27. doi: 10.1186/bcr2257 19432961 PMC2716495

[56] Floros KV Lochmann TL Hu B Monterrubio C Hughes MT Wells JD . Coamplification of miR-4728 protects HER2-amplified breast cancers from targeted therapy. Proc Natl Acad Sci USA. (2018) 115:E2594–603. doi: 10.1073/pnas.1717820115 29476008 PMC5856537

[57] Dang K Myers KA . The role of hypoxia-induced miR-210 in cancer progression. Int J Mol Sci. (2015) 16:6353–72. doi: 10.3390/ijms16036353 25809609 PMC4394536

[58] Gee HE Ivan C Calin GA Ivan M . HypoxamiRs and cancer: from biology to targeted therapy. Antioxid Redox Signal. (2014) 21:1220–38. doi: 10.1089/ars.2013.5639 24111776 PMC4142802

[59] Zhang Y Lv L Zheng R Xie R Yu Y Liao H . Transcriptionally regulated miR-26a-5p may act as BRCAness in triple-negative breast cancer. Breast Cancer Res. (2023) 25:75. doi: 10.1186/s13058-023-01663-y 37365643 PMC10294332

[60] Castellano L Dabrowska A Pellegrino L Ottaviani S Cathcart P Frampton AE . Sustained expression of miR-26a promotes chromosomal instability and tumorigenesis through regulation of CHFR. Nucleic Acids Res. (2017) 45:4401–12. doi: 10.1093/nar/gkx022 28126920 PMC5416844

[61] Silkworth WT Nardi IK Scholl LM Cimini D . Multipolar spindle pole coalescence is a major source of kinetochore mis-attachment and chromosome mis-segregation in cancer cells. PloS One. (2009) 4:e6564. doi: 10.1371/journal.pone.0006564 19668340 PMC2719800

[62] Diederichs S Haber DA . Sequence variations of microRNAs in human cancer: alterations in predicted secondary structure do not affect processing. Cancer Res. (2006) 66:6097–104. doi: 10.1158/0008-5472.CAN-06-0537 16778182

[63] Plantamura I Cosentino G Cataldo A . MicroRNAs and DNA-damaging drugs in breast cancer: Strength in numbers. Front Oncol. (2018) 8:352. doi: 10.3389/fonc.2018.00352 30234015 PMC6129576

[64] Yang S Li D . Role of microRNAs in triple-negative breast cancer and new therapeutic concepts (review). Oncol Lett. (2024) 28:431. doi: 10.3892/ol.2024.14565 39049985 PMC11268089

[65] Saadeldin MK Curigliano G Abdel-Aziz AK . Deciphering the complex interplay of long noncoding RNAs and Aurora kinases: Novel insights into breast cancer development and therapeutic strategies. Future Pharmacol. (2024) 4:466–78. doi: 10.3390/futurepharmacol4030026 30654563

[66] Hannafon BN Cai A Calloway CL Xu YF Zhang R Fung KM . MiR-23b and miR-27b are oncogenic microRNAs in breast cancer: Evidence from a CRISPR/Cas9 deletion study. BMC Cancer. (2019) 19:642. doi: 10.1186/s12885-019-5839-2 31253120 PMC6599331

[67] Peng Y Croce CM . The role of microRNAs in human cancer. Signal Transduct Target Ther. (2016) 1:15004. doi: 10.1038/sigtrans.2015.4 29263891 PMC5661652

[68] Solaimani M Hosseinzadeh S Abasi M . Non-coding RNAs, a double-edged sword in breast cancer prognosis. Cancer Cell Int. (2025) 25:123. doi: 10.1186/s12935-025-03679-0 40170036 PMC11959806

[69] Amirian M Jafari-Nozad AM Darroudi M Farkhondeh T Samarghandian S . Overview of the miR-29 family members' function in breast cancer. BioMed Pharmacother. (2023) 159:114257. doi: 10.1016/j.biopha.2023.114257 36652981

[70] Kwon JJ Factora TD Dey S Kota J . A systematic review of miR-29 in cancer. Mol Ther Oncolytics. (2019) 12:173–94. doi: 10.1016/j.omto.2018.12.011 30788428 PMC6369137

[71] Wu Z Huang X Huang X Zou Q Guo Y . The inhibitory role of miR-29 in growth of breast cancer cells. J Exp Clin Cancer Res. (2013) 32:98. doi: 10.1186/1756-9966-32-98 24289849 PMC4176287

[72] Beňačka R Szabóová D Guľašová Z Hertelyová Z . Non-coding RNAs in breast cancer: Diagnostic and therapeutic implications. Int J Mol Sci. (2025) 26:127. doi: 10.3390/ijms26010127 39795985 PMC11719911

[73] Maleki Dana P Mansournia MA Mirhashemi SM . PIWI-interacting RNAs: New biomarkers for diagnosis and treatment of breast cancer. Cell Biosci. (2020) 10:44. doi: 10.1186/s13578-020-00403-5 32211149 PMC7092456

[74] Cheng Y Wang Q Jiang W Bian Y Zhou Y Gou A . Emerging roles of piRNAs in cancer: Challenges and prospects. Aging. (2019) 11:9932–46. doi: 10.18632/aging.102417 31727866 PMC6874451

[75] Garcia-Silva MR Montenegro S Dacosta S Tosar JP Cayota A . PIWIL1 is recruited to centrosomes during mitosis in colorectal cancer cells and is linked to cell cycle progression. Sci Rep. (2024) 14:23928. doi: 10.1038/s41598-024-75098-6 39397093 PMC11471757

[76] Tan L Mai D Zhang B Jiang X Zhang J Bai R . PIWI-interacting RNA-36712 restrains breast cancer progression and chemoresistance by interaction with SEPW1 pseudogene SEPW1P RNA. Mol Cancer. (2019) 18:9. doi: 10.1186/s12943-019-0940-3 30636640 PMC6330501

[77] Ding X Li Y Lü J Zhao Q Guo Y Lu Z . piRNA-823 is involved in cancer stem cell regulation through altering DNA methylation in association with luminal breast cancer. Front Cell Dev Biol. (2021) 9:641052. doi: 10.3389/fcell.2021.641052 33791297 PMC8005588

[78] Liang J Wen J Huang Z Chen XP Zhang BX Chu L . Small nucleolar RNAs: Insight into their function in cancer. Front Oncol. (2019) 9:587. doi: 10.3389/fonc.2019.00587 31338327 PMC6629867

[79] Lorenzo HK . Small nucleolar RNAs as emerging players in cancer biology and precision medicine. Cancers (Basel). (2025) 17:3847. doi: 10.3390/cancers17233847 41375049 PMC12691191

[80] Han C Sun LY Luo XQ Pan Q Sun YM Zeng ZC . Chromatin-associated orphan snoRNA regulates DNA damage-mediated differentiation via a non-canonical complex. Cell Rep. (2022) 38:110421. doi: 10.1016/j.celrep.2022.110421 35354054

[81] Li X Zhao X Xie L Song X Song X . Identification of four snoRNAs (SNORD16, SNORA73B, SCARNA4, and SNORD49B) as novel non-invasive biomarkers for diagnosis of breast cancer. Cancer Cell Int. (2024) 24:55. doi: 10.1186/s12935-024-03237-0 38311725 PMC10840236

[82] Li JN Loh ZJ Chen HW Lee IY Tsai JH Chen PS . SnoRNA U50A mediates everolimus resistance in breast cancer through mTOR downregulation. Transl Oncol. (2024) 48:102062. doi: 10.1016/j.tranon.2024.102062 39094511 PMC11342273

[83] Li S Jin Z Song X Ma J Peng Z Yu H . The small nucleolar RNA SNORA51 enhances breast cancer stem cell-like properties via the RPL3/NPM1/c-MYC pathway. Mol Carcinog. (2024) 63:1117–32. doi: 10.1002/mc.23713 38421204

[84] Su X Feng C Wang S Shi L Gu Q Zhang H . The noncoding RNAs SNORD50A and SNORD50B-mediated TRIM21-GMPS interaction promotes the growth of p53 wild-type breast cancers by degrading p53. Cell Death Differ. (2021) 28:2450–64. doi: 10.1038/s41418-021-00762-7 33742136 PMC8329294

[85] Gong J Li Y Liu CJ Xiang Y Li C Ye Y . A pan-cancer analysis of the expression and clinical relevance of small nucleolar RNAs in human cancer. Cell Rep. (2017) 21:1968–81. doi: 10.1016/j.celrep.2017.10.070 29141226

[86] Siprashvili Z Webster DE Johnston D Shenoy RM Ungewickell AJ Bhaduri A . The noncoding RNAs SNORD50A and SNORD50B bind K-Ras and are recurrently deleted in human cancer. Nat Genet. (2016) 48:53–8. doi: 10.1038/ng.3452 26595770 PMC5324971

[87] Chen B Dragomir MP Fabris L Bayraktar R Knutsen E Liu X . The long noncoding RNA CCAT2 induces chromosomal instability through BOP1-AURKB signaling. Gastroenterology. (2020) 159:2146–2162.e33. doi: 10.1053/j.gastro.2020.08.059 32805281 PMC7725986

[88] Pan H Wang H Zhang X Yang F Fan X Zhang H . Chromosomal instability-associated MAT1 lncRNA insulates MLL1-guided histone methylation and accelerates tumorigenesis. Cell Rep. (2022) 41:111829. doi: 10.1016/j.celrep.2022.111829 36516779

[89] Liu L Zhang Y Lu J . The roles of long noncoding RNAs in breast cancer metastasis. Cell Death Dis. (2020) 11:749. doi: 10.1038/s41419-020-02954-4 32929060 PMC7490374

[90] Chen Q Chen H Yang H Tao Y Zhu X . Characteristics of chromosomal instability-related lncRNAs associated with progression and prognosis in breast cancer. Mol Med Rep. (2026) 33:124. doi: 10.3892/mmr.2026.13834 41789582 PMC12994133

[91] Andonegui-Elguera MA Cáceres-Gutiérrez RE Oliva-Rico D Díaz-Chávez J Herrera LA . LncRNAs-associated to genomic instability: a barrier to cancer therapy effectiveness. Front Genet. (2022) 13:984329. doi: 10.3389/fgene.2022.984329 36479250 PMC9719945

[92] Carter SL Eklund AC Kohane IS Harris LN Szallasi Z . A signature of chromosomal instability inferred from gene expression profiles predicts clinical outcome in multiple human cancers. Nat Genet. (2006) 38:1043–8. doi: 10.1038/ng1861 16921376

[93] Winkle M El-Daly SM Fabbri M Calin GA . Noncoding RNA therapeutics—challenges and potential solutions. Nat Rev Drug Discov. (2021) 20:629–51. doi: 10.1038/s41573-021-00219-z 34145432 PMC8212082

[94] Tracy KM Tye CE Ghule PN Malaby HLH Stumpff J Stein JL . Mitotically-associated lncRNA (MANCR) affects genomic stability and cell division in aggressive breast cancer. Mol Cancer Res. (2018) 16:587–98. doi: 10.1158/1541-7786.MCR-17-0548 29378907 PMC5882506

[95] Sharma V Khurana S Kubben N Abdelmohsen K Oberdoerffer P Gorospe M . A BRCA 1‐interacting lnc RNA regulates homologous recombination. EMBO Rep. (2015) 16:1520–34. doi: 10.15252/embr.201540437 26412854 PMC4641504

[96] Luo R Wu J Chen X Liu Y Liu D Song E . ATR/Chk1 interacting lncRNA modulates DNA damage response to induce breast cancer chemoresistance. Cell Insight. (2024) 3:100183. doi: 10.1016/J.CELLIN.2024.100183 39148723 PMC11325286

[97] Alves-Vale C Capela AM Tavares-Marcos C Domingues-Silva B Pereira B Santos F . Expression of NORAD correlates with breast cancer aggressiveness and protects breast cancer cells from chemotherapy. Mol Ther Nucleic Acids. (2023) 33:910–24. doi: 10.1016/j.omtn.2023.08.019 37680988 PMC10480464

[98] Zhou K Ou Q Wang G Zhang W Hao Y Li W . High long non-coding RNA NORAD expression predicts poor prognosis and promotes breast cancer progression by regulating TGF-β pathway. Cancer Cell Int. (2019) 19:63. doi: 10.1186/s12935-019-0781-6 30930692 PMC6425604

[99] Zhang Q Wang X Shao Z Zhang Y Zhang L Chen M . LINC01235 promotes clonal evolution through DNA replication licensing-induced chromosomal instability in breast cancer. Adv Sci. (2025) 12:e2413527. doi: 10.1002/advs.202413527 39950924 PMC11984920

[100] Volovat SR Volovat C Hordila I Hordila DA Mirestean CC Miron OT . MiRNA and lncRNA as potential biomarkers in triple-negative breast cancer: a review. Front Oncol. (2020) 10:526850. doi: 10.3389/fonc.2020.526850 33330019 PMC7716774

[101] Hajibabaei S Nafissi N Azimi Y Mahdian R Rahimi-Jamnani F Valizadeh V . Targeting long non-coding RNA MALAT1 reverses cancerous phenotypes of breast cancer cells through microRNA-561-3p/TOP2A axis. Sci Rep. (2023) 13:8652. doi: 10.1038/s41598-023-35639-x 37244966 PMC10224942

[102] Betts JA Moradi Marjaneh M Al-Ejeh F Lim YC Shi W Sivakumaran H . Long noncoding RNAs CUPID1 and CUPID2 mediate breast cancer risk at 11q13 by modulating the response to DNA damage. Am J Hum Genet. (2017) 101:255–66. doi: 10.1016/j.ajhg.2017.07.007 28777932 PMC5544418

[103] Niknafs YS Han S Ma T Speers C Zhang C Wilder-Romans K . The lncRNA landscape of breast cancer reveals a role for DSCAM-AS1 in breast cancer progression. Nat Commun. (2016) 7:12791. doi: 10.1038/ncomms12791 27666543 PMC5052669

[104] Zhang M Wang N Song P Fu Y Ren Y Li Z . LncRNA GATA3-AS1 facilitates tumour progression and immune escape in triple-negative breast cancer through destabilization of GATA3 but stabilization of PD-L1. Cell Prolif. (2020) 53:e12855. doi: 10.1111/cpr.12855 32687248 PMC7507373

[105] Gupta RA Shah N Wang KC Kim J Horlings HM Wong DJ . Long non-coding RNA HOTAIR reprograms chromatin state to promote cancer metastasis. Nature. (2010) 464:1071–6. doi: 10.1038/nature08975 20393566 PMC3049919

[106] Cedro-Tanda A Ríos-Romero M Romero-Córdoba S Cisneros-Villanueva M Rebollar-Vega RG Alfaro-Ruiz LA . A lncRNA landscape in breast cancer reveals a potential role for AC009283.1 in proliferation and apoptosis in HER2-enriched subtype. Sci Rep. (2020) 10:13146. doi: 10.1038/s41598-020-69905-z 32753692 PMC7403317

[107] Gutschner T Hämmerle M Diederichs S . MALAT1—a paradigm for long noncoding RNA function in cancer. J Mol Med (Berl). (2013) 91:791–801. doi: 10.1007/s00109-013-1028-y 23529762

[108] Lu G Li Y Ma Y Lu J Chen Y Jiang Q . Long noncoding RNA LINC00511 contributes to breast cancer tumourigenesis and stemness by inducing the miR-185-3p/E2F1/Nanog axis. J Exp Clin Cancer Res. (2018) 37:289. doi: 10.1186/s13046-018-0945-6 30482236 PMC6260744

[109] Zhang J Sui S Wu H Zhang J Zhang X Xu S . The transcriptional landscape of lncRNAs reveals the oncogenic function of LINC00511 in ER-negative breast cancer. Cell Death Dis. (2019) 10:599. doi: 10.1038/s41419-019-1835-3 31395854 PMC6687715

[110] Jiao Y Li S Wang X Yi M Wei H Rong S . A genomic instability-related lncRNA model for predicting prognosis and immune checkpoint inhibitor efficacy in breast cancer. Front Immunol. (2022) 13:929846. doi: 10.3389/fimmu.2022.929846 35990656 PMC9389369

[111] Li X Jin F Li Y . A novel autophagy-related lncRNA prognostic risk model for breast cancer. J Cell Mol Med. (2021) 25:4–14. doi: 10.1111/jcmm.15980 33216456 PMC7810925

[112] Li B Liu D Xu J Lu Z Liu Q Zhao X . Overexpression of lncRNA MAPT-AS1 exacerbates cell proliferation and metastasis in breast cancer. Transl Cancer Res. (2022) 11:835–47. doi: 10.21037/tcr-22-719 35571636 PMC9091000

[113] Pan Y Pan Y Cheng Y Yang F Yao Z Wang O . Knockdown of lncRNA MAPT-AS1 inhibits proliferation and migration and sensitizes cancer cells to paclitaxel by regulating MAPT expression in ER-negative breast cancers. Cell Biosci. (2018) 8:7. doi: 10.1186/s13578-018-0207-5 29441192 PMC5799917

[114] Zhang X Zhang H Li J Ma X He Z Liu C . 6-lncRNA assessment model for monitoring and prognosis of HER2-positive breast cancer: based on transcriptome data. Pathol Oncol Res. (2021) 27:609083. doi: 10.3389/pore.2021.609083 34257572 PMC8262145

[115] Qian W Zhu Y Wu M Guo Q Wu Z Lobie PE . Linc00668 promotes invasion and stem cell-like properties of breast cancer cells by interaction with SND1. Front Oncol. (2020) 10:88. doi: 10.3389/fonc.2020.00088 32117742 PMC7033544

[116] Giannakakis A Tsifintaris M Gouzouasis V Ow GS Aau MY Papp C . KDM7A-DT induces genotoxic stress, tumorigenesis, and progression of p53 missense mutation-associated invasive breast cancer. Front Oncol. (2024) 14:1227151. doi: 10.3389/fonc.2024.1227151 38756663 PMC11097164

[117] Arjumand W Asiaf A Ahmad ST . Noncoding RNAs in DNA damage response: Opportunities for cancer therapeutics. In: Methods in Molecular Biology, vol. 1699. (New York, NY) (2018). p. 3–21. doi: 10.1007/978-1-4939-7435-1_1 29086365

[118] Slack FJ Chinnaiyan AM . The role of non-coding RNAs in oncology. Cell. (2019) 179:1033–55. doi: 10.1016/j.cell.2019.10.017 31730848 PMC7347159

[119] Saliminejad K Khorram Khorshid HR Soleymani Fard S Ghaffari SH . An overview of microRNAs: Biology, functions, therapeutics, and analysis methods. J Cell Physiol. (2019) 234:5451–65. doi: 10.1002/jcp.27486 30471116

[120] Diener C Keller A Meese E . Emerging concepts of miRNA therapeutics: from cells to clinic. Trends Genet. (2022) 38:613–26. doi: 10.1016/j.tig.2022.02.006 35303998

[121] Menon A Abd-Aziz N Khalid K Poh CL Naidu R . miRNA: A promising therapeutic target in cancer. Int J Mol Sci. (2022) 23:11502. doi: 10.3390/ijms231911502 36232799 PMC9569513

[122] Pandey S Yadav P . Exploring the therapeutic potential of microRNAs: targeted gene regulation strategies for enhanced cancer therapy. J Genet Eng Biotechnol. (2025) 23:100556. doi: 10.1016/j.jgeb.2025.100556 41386822 PMC12405681

[123] Rupaimoole R Slack FJ . MicroRNA therapeutics: Towards a new era for the management of cancer and other diseases. Nat Rev Drug Discov. (2017) 16:203–22. doi: 10.1038/nrd.2016.246 28209991

[124] Normann LS Haugen MH Aure MR Kristensen VN Mælandsmo GM Sahlberg KK . miR-101-5p acts as a tumor suppressor in HER2-positive breast cancer cells and improves targeted therapy. Breast Cancer: Targets Ther. (2022) 14:25–39. doi: 10.2147/BCTT.S338404 35256859 PMC8898020

[125] Farheen J Hosmane NS Zhao R Zhao Q Iqbal MZ Kong X . Nanomaterial-assisted CRISPR gene-engineering – a hallmark for triple-negative breast cancer therapeutics advancement. Mater Today Bio. (2022) 16:100450. doi: 10.1016/j.mtbio.2022.100450 36267139 PMC9576993

[126] Liu X Zhang G Yu T He J Liu J Chai X . Exosomes deliver lncRNA DARS-AS1 siRNA to inhibit chronic unpredictable mild stress-induced TNBC metastasis. Cancer Lett. (2022) 543:215781. doi: 10.1016/j.canlet.2022.215781 35688263

[127] Toden S Zumwalt TJ Goel A . Non-coding RNAs and potential therapeutic targeting in cancer. Biochim Biophys Acta Rev Cancer. (2021) 1875:188491. doi: 10.1016/j.bbcan.2020.188491 33316377 PMC7856203

[128] Zhang Y Fan X Hong J Yang E Xuan C Fang H . Diagnostic implications of lncRNA NORAD in breast cancer. Sci Rep. (2023) 13:20426. doi: 10.1038/s41598-023-47434-9 37993524 PMC10665357

[129] Aboutalebi Vand Beilankouhi E Sanaat Z Hosseinpour Feizi MA Mehdizadeh A Safaralizadeh R . Investigation of circulating miR-182-3p, miR -382-3p and miR -93, miR -142-3p involved in tamoxifen resistance and sensitivity in luminal-subtype breast cancer patients: a case–control study. Naunyn Schmiedebergs Arch Pharmacol. (2025) 398:7505–16. doi: 10.1007/s00210-024-03770-9 39754680

[130] Patellongi I Amiruddin A Massi MN Islam AA Pratama MY Sutandyo N . Circulating miR-221/222 expression as microRNA biomarker predicting tamoxifen treatment outcome: a case–control study. Ann Med Surg. (2023) 85:3806–15. doi: 10.1097/ms9.0000000000001061 37554919 PMC10406100

[131] Carvalho E Schmitt F Vale N . Interplay between microRNAs and breast cancer therapies: personalized therapeutic potential for HER2-low breast cancer. Cancers (Basel). (2025) 17:3672. doi: 10.3390/cancers17223672 41301038 PMC12651918

[132] He X Lin F Jia R Xia Y Liang Z Xiao X . Coordinated modulation of long non-coding RNA ASBEL and curcumin co-delivery through multicomponent nanocomplexes for synchronous triple-negative breast cancer theranostics. J Nanobiotechnol. (2023) 21:397. doi: 10.1186/s12951-023-02168-8 37904215 PMC10617238

[133] Ouyang B Bi M Jadhao M Bick G Zhang X . miR-205 regulates tamoxifen resistance by targeting estrogen receptor coactivator MED1 in human breast cancer. Cancers (Basel). (2024) 16:3992. doi: 10.3390/cancers16233992 39682180 PMC11640040

[134] Yu Y Yin W Yu ZH Zhou YJ Chi JR Ge J . MiR-190 enhances endocrine therapy sensitivity by regulating SOX9 expression in breast cancer. J Exp Clin Cancer Res. (2019) 38:22. doi: 10.1186/s13046-019-1039-9 30658681 PMC6339391

[135] Arce C Perez-Plasencia C Gonzalez-Fierro A de la Cruz-Hernández E Revilla-Vazquez A Chávez-Blanco A . A proof-of-principle study of epigenetic therapy with hydralazine and magnesium valproate plus doxorubicin cyclophosphamide as neoadjuvant therapy for locally advanced breast cancer. BMC Cancer. (2007) 7:A23. doi: 10.1186/1471-2407-7-S1-A23 38164791

[136] Tan WW Allred JB Moreno-Aspitia A Northfelt DW Ingle JN Goetz MP . Phase I study of panobinostat (LBH589) and letrozole in postmenopausal metastatic breast cancer patients. Clin Breast Cancer. (2016) 16:82–6. doi: 10.1016/j.clbc.2015.11.003 26774555 PMC5567753

[137] Connolly RM Li H Jankowitz RC Zhang Z Rudek MA Jeter SC . Combination epigenetic therapy in advanced breast cancer with 5-azacitidine and entinostat: a phase II National Cancer Institute/Stand Up to Cancer study. Clin Cancer Res. (2017) 23:2691–701. doi: 10.1158/1078-0432.CCR-16-1729 27979916 PMC5457329

[138] Zhang Q Wang T Geng C Zhang Y Zhang J Ning Z . Exploratory clinical study of chidamide, an oral subtype-selective histone deacetylase inhibitor, in combination with exemestane in hormone receptor-positive advanced breast cancer. Chin J Cancer Res. (2018) 30:605–12. doi: 10.21147/j.issn.1000-9604.2018.06.05 30700929 PMC6328505

[139] Vernier M McGuirk S Dufour CR Scholtes C Li X Bourmeau G . Inhibition of DNMT1 and ERRα crosstalk suppresses breast cancer via derepression of IRF4. Oncogene. (2020) 39:6406–20. doi: 10.1038/s41388-020-01438-1 32855526 PMC7544553

[140] Zucchetti B Shimada AK Katz A Curigliano G . The role of histone deacetylase inhibitors in metastatic breast cancer. Breast. (2019) 43:130–4. doi: 10.1016/j.breast.2018.12.001 30553187

[141] Esteller M . Epigenetics in cancer. N Engl J Med. (2008) 358:1148–59. doi: 10.1056/NEJMra072067 18337604

[142] Saito Y Liang G Egger G Friedman JM Chuang JC Coetzee GA . Specific activation of microRNA-127 with downregulation of the proto-oncogene BCL6 by chromatin-modifying drugs in human cancer cells. Cancer Cell. (2006) 9:435–43. doi: 10.1016/j.ccr.2006.04.020 16766263

[143] Prabhakaran R Thamarai R Sivasamy S Dhandayuthapani S Batra J Kamaraj C . Epigenetic frontiers: miRNAs, long non-coding RNAs and nanomaterials are pioneering to cancer therapy. Epigenet Chromatin. (2024) 17:31. doi: 10.1186/s13072-024-00554-6 39415281 PMC11484394

[144] Muñoz JP Pérez-Moreno P Pérez Y Calaf GM . The role of microRNAs in breast cancer and the challenges of their clinical application. Diagnost (Basel). (2023) 13:3072. doi: 10.3390/diagnostics13193072 37835815 PMC10572677

[145] Anastasiadou E Jacob LS Slack FJ . Non-coding RNA networks in cancer. Nat Rev Cancer. (2018) 18:5–18. doi: 10.1038/nrc.2017.99 29170536 PMC6337726

